# Transcriptomic Profiling Reveals Differentially Expressed Genes Associated with Pine Wood Nematode Resistance in Masson Pine (*Pinus massoniana* Lamb.)

**DOI:** 10.1038/s41598-017-04944-7

**Published:** 2017-07-05

**Authors:** Qinghua Liu, Yongcheng Wei, Liuyi Xu, Yanping Hao, Xuelian Chen, Zhichun Zhou

**Affiliations:** 1 Research Institute of Subtropical Forestry, Chinese Academy of Forestry, Hangzhou, Zhejiang People’s Republic of China; 2 Zhejiang Provincial Key Laboratory of Tree Breeding, Hangzhou, Zhejiang People’s Republic of China; 3 Anhui Academy of Forestry, Hefei, Anhui People’s Republic of China

## Abstract

Pine wilt disease caused by pine wood nematode (*Bursaphelenchus xylophilus*, PWN) is a severe forest disease of the genus *Pinus*. Masson pine as an important timber and oleoresin resource in South China, is the major species infected by pine wilt disease. However, the underlying mechanism of pine resistance is still unclear. Here, we performed a transcriptomics analysis to identify differentially expressed genes associated with resistance to PWN infection. By comparing the expression profiles of resistant and susceptible trees inoculated with PWN at 1, 15, or 30 days post-inoculation (dpi), 260, 371 and 152 differentially expressed genes (DEGs) in resistant trees and 756, 2179 and 398 DEGs in susceptible trees were obtained. Gene Ontology enrichment analysis of DEGs revealed that the most significant biological processes were “syncytium formation” in the resistant phenotype and “response to stress” and “terpenoid biosynthesis” in the susceptible phenotype at 1 and 15 dpi, respectively. Furthermore, some key DEGs with potential regulatory roles to PWN infection, including expansins, pinene synthases and reactive oxidation species (ROS)-related genes were evaluated in detail. Finally, we propose that the biosynthesis of oleoresin and capability of ROS scavenging are pivotal to the high resistance of PWN.

## Introduction

Pine wilt disease was first reported in Nagasaki City, Japan, in 1905 as a type of severe forest disease affecting the genus *Pinus* ^1^. Subsequently, pine wilt disease has also been discovered in America, Canada, Mexico, Korea, China and Portugal^2–4^. This disease is mainly caused by the pine wood nematode (PWN) (*Bursaphelenchus xylophilus*) through its vector of cerambycid beetles (*Monochamus* spp.) in the course of feeding on twigs of the genus *Pinus*, such as *Pinus thunbergii*, *P. densiflora*, *P. massoniana*, *P. pinaster* and *P. elliottii* ^4–6^. It has caused enormous economic losses and threatened the ecosystems of pine forest in many countries, especially in Japan and China. Masson pine (*P. massoniana* Lamb.) is a commercially important conifer for timber and oleoresin in South China. The natural distribution extends from 21°41′N to 33°56′N and 102°10′E to 123°14′E and covers 17 administrative provinces. In China, masson pine is the main tree species damaged by pine wilt disease. Since pine wilt disease was first found in Nanjing City, China, in 1982, the disease has been spread to 15 administrative provinces according to the announcement from China’s State Forestry Administration in 2013, where approximately 5,000,000 m^3^ of masson pines have been damaged by PWN according to incomplete statistical records. Furthermore, the number of damaged masson pines is increasing every year.

Forestry managers and researchers have been searching for methods to control pine wilt disease. Some control measures, such as removing the dead trees, spraying pesticide to kill the vector of cerambycidae, and constructing mixed forests, only delay the spread speed of pine wilt disease^7^ and cannot control the disease completely. Notably, not all pine trees die after they are invaded by PWN, and resistant trees exist within and among populations of the genus *Pinus* ^8–10^. Therefore, breeding resistant pine trees might be the most economical and effective control measure, and this method has been used in many countries, such as Japan, China, and Portugal^10–12^. The project of breeding resistance to pine wilt disease has been ongoing since 1978 in Japan. The average survival rate of open-pollinated progeny from selected resistant trees of *P. densiflora* and *P. thunbergii* was 16% and 40% higher than from unselected populations, respectively^12^. In China, the breeding project of resistance to pine wilt disease began in Anhui province in 2001. At present, 1201 resistant individuals were selected from masson pines^10^, which provided valuable resources for understanding the mechanism of resistance to PWN.

The anatomical and biochemical changes of pine trees with PWN infection have been characterized over the past 40 years^13^. As for the pathogenic mechanism of pine wilt disease, several hypotheses have been put forward, such as cavitation formed by terpenoids blocking water transport in xylem tracheids^14, 15^, phytotoxins produced by PWN-associated bacteria causing the death of pines^16, 17^, the cellulose and pectin secreted by PWN degrading the cell walls of pines and causing their death^18^, etc. Based on previous studies, resistant trees can control PWN infestation effectively by stopping nematode migration in the pine trees or preventing the tissues of the cortex, phloem, cambium and resin canals to be severely destructed^19^. Several studies have characterized the resistance to pine wood nematode infection at the molecular level in *P. thunbergii* and *P. densiflora*. By using suppression subtractive hybridization, it has been proposed that the pathogenesis-related genes and cell wall-related genes induced by reactive oxygen species are crucial in the defense against PWN infestation^20–22^. However, different pine species have different defense mechanisms against PWN^23^. Compared with *P. thunbergii*, *P. massoniana* was more resistant to PWN^24^. Until now, only a few studies on the resistance mechanism of masson pine against PWN at the transcriptome level have been reported. Xu *et al*.^25^ demonstrated transcriptional responses to PWN infection in non-resistant masson pines. However, the successful defense mechanism against PWN remains unclear in masson pine.

Next-generation high-throughput sequencing is a powerful tool to identify differential transcripts from whole genome under different conditions. In this study, this method was used to characterize the difference in the transcript profiles of resistant and susceptible pines after PWN inoculation. Based on the results from the previous year, we found that the needles began to turn yellow at 30 days after the masson pines were inoculated with PWN. The resistant and susceptible trees were sampled at 1, 15 and 30 dpi. The goal was 1) to understand the different responsive mechanisms of resistant and susceptible phenotypes by identifying their differentially expressed genes after PWN inoculation compared to a water control; and 2) to elucidate the successful defense mechanism against PWN in the resistant phenotype by identifying the differentially expressed genes of the resistant and susceptible phenotypes after PWN inoculation. We found that a small number of DEGs were induced by PWN in resistant trees compared to susceptible trees at each time point. Detailed gene expression analysis revealed that expansin, monoterpene synthase (mono-TPS), sequi-TPS and reactive oxidation species (ROS)-related genes were associated with resistance to PWN infection. We observed that the endogenous capability of oleoresin biosynthesis and ROS scavenging was essential to the resistance to PWN, which may be the key breeding characteristics of highly resistant pine trees. This work provides comprehensive gene resources underlying PWN tolerance and could facilitate the genetic breeding process of valuable masson pines.

## Results

### Histological characterizations of resistant and susceptible clones in masson pine and transcriptome assembly

**Figure 1:**
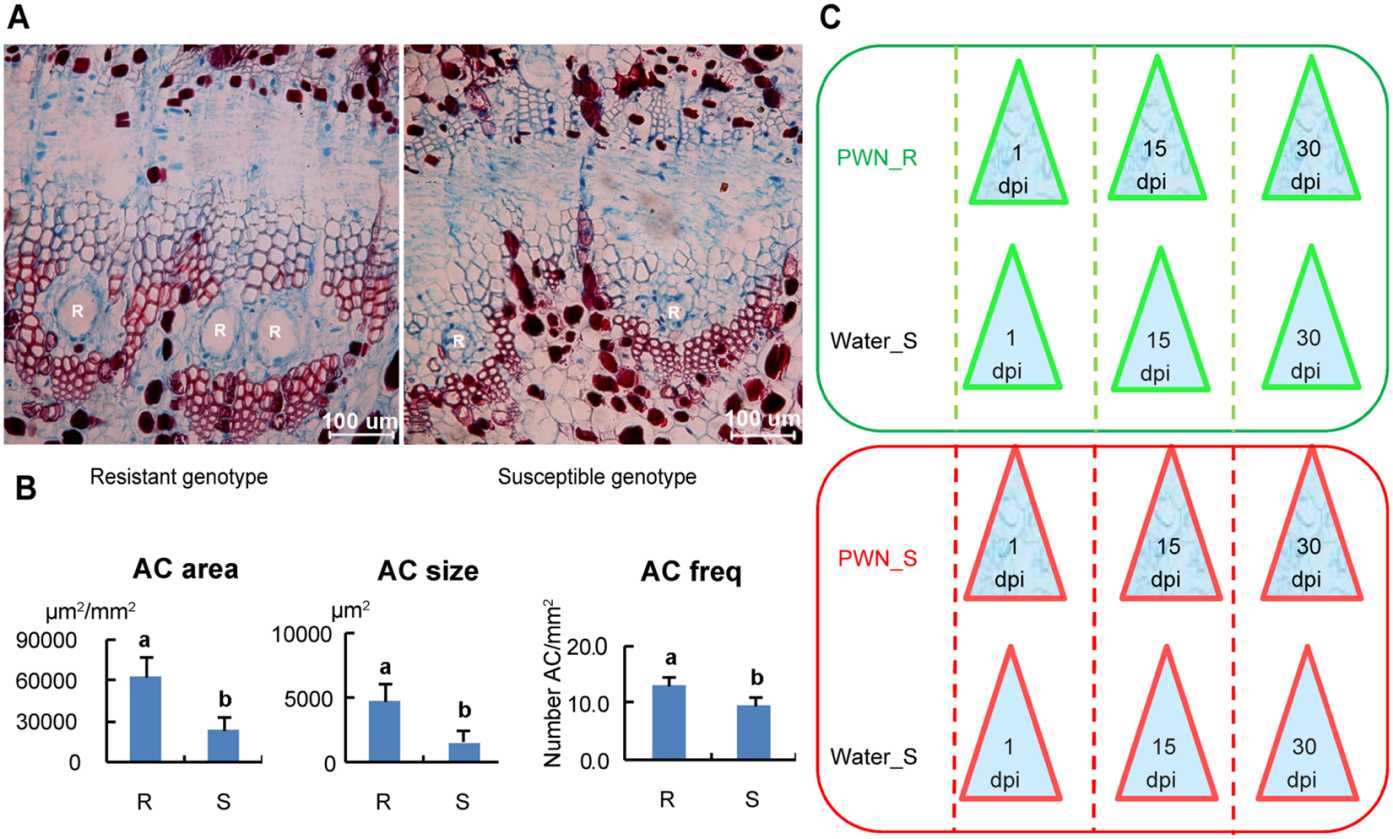
Resistant and susceptible phenotypes used in this study and their symptoms after PWN inoculation. (**A**) Vertical resin ducts in the cross section of the stem for resistant and susceptible phenotypes; the bar is 100 μm. (**B**) Difference in the parameters of the resin ducts between resistant and susceptible phenotypes in which resistant phenotypes had a larger AC size (resin duct size), AC area (resin duct area) and AC freq (resin duct frequency) than the susceptible phenotype (AC size comparison: n = 30, p = 0.001; AC area comparison: n = 30, p = 0.003; AC freq comparison: n = 30, p = 0.009). The mean + SE is represented by each bar. Different letters “a” and “b” on top of bars indicate significant differences (*t*-test, α = 0.05). (**C**) The materials used for transcriptome sequencing and each treatment contained three biological replicates. R: resin canal; X: xylem; P: pith; nX: new xylem.

To understand the molecular basis of nematode response in masson pine, 2 clones of masson pine that displayed distinctive phenotypes (resistant and susceptible) after PWN inoculation were identified. Before inoculating PWN, vertical resin duct characteristics of the shoot were compared between resistant and susceptible phenotypes (Fig. 1A). The result showed that resin duct size, area and number per unit area in cross section were all significantly different (P < 0.05), and the resistant phenotype had a larger resin duct size, area and number (Fig. 1B). We performed next-generation sequencing to generate the transcriptome of a stem of masson pine. After quality assessment and data filtering, we obtained 48,316,242 high quality reads (9.7 gigabase pairs), with 85.25% bases over Q30. Clean reads were assembled into 132,648 transcripts, with a mean length of 904 bp by Trinity (Supplementary Table S1). Finally, 80,340 unigenes were obtained through the clustering of overlapped transcripts with a mean length of 675 bp. Among these unigenes, 14,994 unigenes (18.66%) were larger than 1 kb. The length distributions of transcripts and unigenes (Supplementary Table S1) have a similar tendency, with N50 values of 1,634 bp and 1,245 bp, respectively. The coefficients of correlation between the 36 samples ranged from 0.74 to 1.00 (Supplementary Table S2).

Functional annotation of the unigenes was carried out by aligning the sequence similarity with several public databases, and the annotation results are listed in Supplementary Table S3. A total of 50,227 (62.5%) unigenes were annotated in the public databases (Supplementary Table S4). Based on the annotation, 32,611 unigenes were distributed into one or more Gene Ontology (GO) terms (Supplementary Fig. S1). In addition, 16,489 sequences could be assigned to the Cluster of Orthologous Groups of proteins (COG) database for functional prediction and classification (Supplementary Fig. S2), and 10,259 of 80,340 unigenes were annotated into 119 pathways according to the Kyoto Encyclopedia of Genes and Genomes (KEGG). These results suggested that the high-quality transcriptome was appropriate for further analyses.

### Transcriptomics profiling in response to nematode inoculation of masson pine

To isolate genes responsive to PWN infection in susceptible and resistant phenotypes of masson pines, 36 sequencing libraries were constructed under two inoculated conditions (nematodes and sterile water) at three time points of 1, 15 and 30 days post-inoculation (dpi), and each sample contained three biological replicates (Fig. 1C). At 30 dpi, a characterization of the resistant trees showed almost no obvious change, but the needles began to turn yellow at 30 dpi, and the trees died at 50 dpi in the susceptible masson pine (Supplementary Fig. S3). In total, we generated 9.00–12.82 million high-quality reads per library. Subsequently, the reads were mapped to the transcriptome database described above. Approximately 80.47–85.87% of the reads matched the sequences in the transcriptome (Supplementary Table S5).

**Figure 2:**
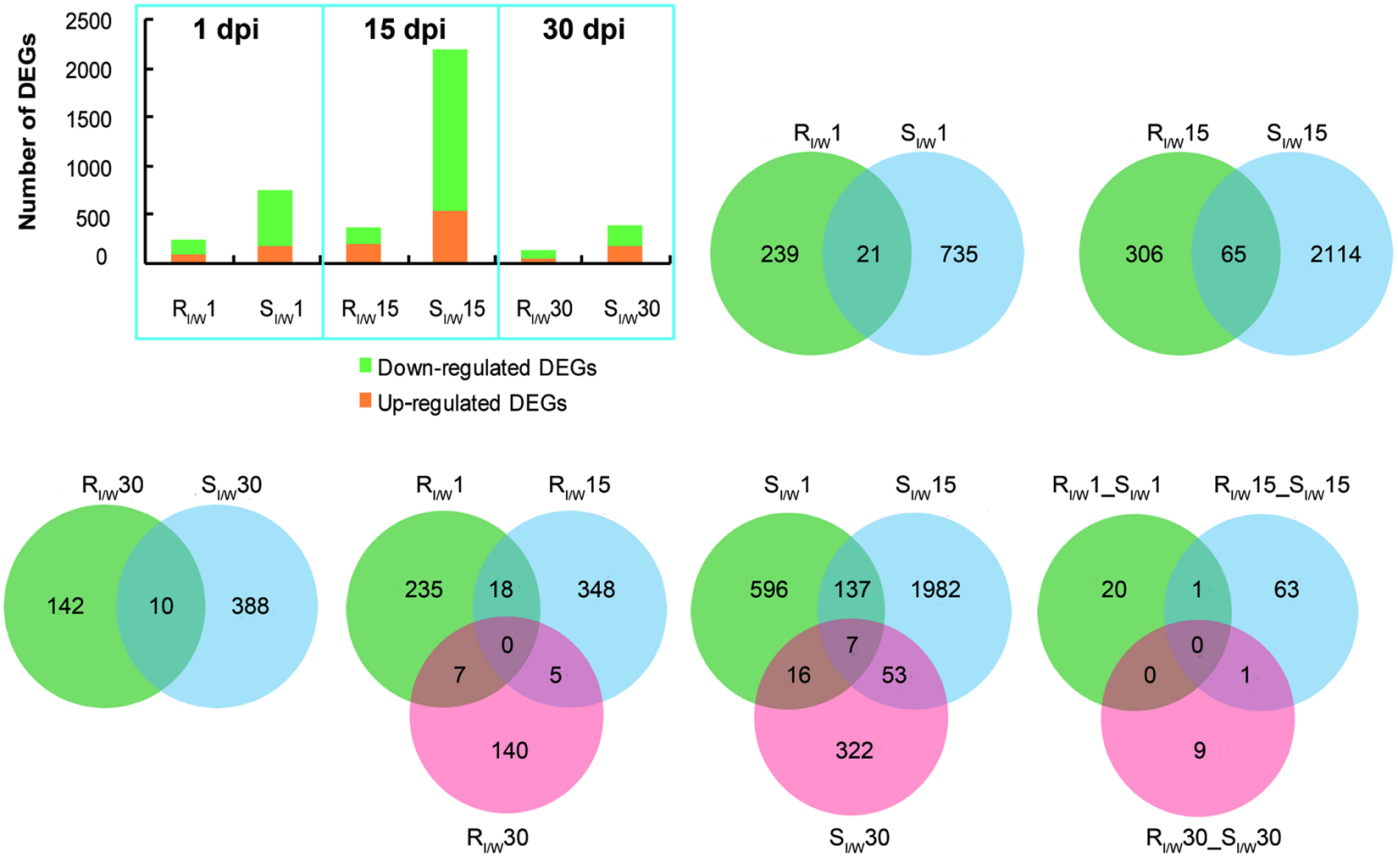
Comparisons of DEGs between two phenotypes at three points. Number of DEGs obtained in resistant and susceptible phenotypes at 1, 15 and 30 dpi and Venn diagram depicting the number and overlapping relationships of DEGs between different phenotypes at the same time point or same phenotype at different time points. R: resistant phenotype; S: susceptible phenotype; I: inoculating PWN; W: inoculating water.

To explore the global difference in the defense responses of susceptible and resistant phenotypes, trees inoculated with sterile water were used as controls in each phenotype, and significant DEGs were compared by applying a cutoff of a false discovery rate (FDR) less than 0.01 and a fold change no less than 2. The obtained DEGs were 260, 371 and 152 in resistant trees and 756, 2179 and 398 in susceptible trees at 1, 15 and 30 dpi, respectively (Fig. 2). At each time point, the number of DEGs in susceptible trees was consistently higher than in resistant trees, suggesting that more DEGs were induced by PWN in susceptible trees than in resistant trees. We also found that the number of DEGs was highest at 15 dpi and least at 30 dpi in both resistant and susceptible trees, and more DEGs were down-regulated in the susceptible phenotype at the three time points.

Between different phenotypes at the same time point, or different time points for each phenotype, there were small numbers of common DEGs (Fig. 2). For example, only 21 common DEGs were found between resistant and susceptible trees at 1 dpi, and 5 common DEGs were found between 15 and 30 dpi in resistant trees, indicating that the defense responses of resistant and susceptible trees might vary as the phenotype and disease progresses.

### Functional enrichment analysis of DEGs

To gain a functional perspective of the genes related to resistance to PWN, we performed a GO enrichment analysis of these DEGs in resistant and susceptible phenotypes at three time points. Approximately 41.45% to 52.56% of DEGs were annotated by the GO database (Supplementary Table S6). The 6 DEG sets underwent GO enrichment analysis to identify over-representative GO terms. We found that in the biological processes category, the fewer enriched GO terms were in the resistant phenotype than in the susceptible phenotype at 1 dpi and 15 dpi, indicating that more biological processes were interfered with in the susceptible phenotype (Table 1). Notably, the enriched GO term “syncytium formation” (a process that was related to the colonization of PWN in plant species) was identified in the resistant phenotype but not in the susceptible phenotype at 1 dpi and 15 dpi, although the unigenes contributing to the GO term were not the same at these two time points (Table 2). However, the most significantly enriched GO terms were “response to stress” for the susceptible phenotype at 1 dpi and 15 dpi. The results indicated that the PWN responses in the two phenotypes were fundamentally different. In biological processes, significantly enriched GO terms involved in the biosynthesis of terpenoid were also observed in the susceptible phenotype at 15 dpi, such as “geranyl diphosphate metabolic process,” “alpha-pinene biosynthetic process” and “monoterpene biosynthetic process,” suggesting that terpenoids might play an important role in the susceptible phenotype. At 30 dpi, the most significant GO terms were “amino acid catabolic process to alcohol via Ehrlich pathway,” “ethanol biosynthetic process involved in glucose fermentation to ethanol” and “NADH oxidation” in the resistant phenotype. However, the only significant GO term was “cellular glucan metabolic process” in the susceptible phenotype at 30 dpi.

**Table 1:** Enriched biological processes in DEGs by GO annotation for resistant and susceptible phenotypes at three points.

Phenotype	Dpi	GO ID	Gene_ontology_term of biological process	P-value	Corrected_P-value
R_I/W_	1	GO:0006949	Syncytium formation	8.25E-07	1.39E-04
	1	GO:0006334	Nucleosome assembly	8.36E-06	1.41E-03
	15	GO:0006949	Syncytium formation	1.67E-04	4.48E-02
	15	GO:1901700	Response to oxygen-containing compound	1.80E-04	4.84E-02
	30	GO:0000947	Amino acid catabolic process to alcohol via Ehrlich pathway	2.16E-05	1.75E-03
	30	GO:0043458	Ethanol biosynthetic process involved in glucose fermentation to ethanol	3.76E-05	3.05E-03
	30	GO:0006116	NADH oxidation	5.18E-05	4.20E-03
	30	GO:0009628	Response to abiotic stimulus	6.75E-05	5.47E-03
	30	GO:0010286	Heat acclimation	1.50E-04	1.22E-02
	30	GO:0051260	Protein homooligomerization	2.08E-04	1.68E-02
S_I/W_	1	GO:0006950	Response to stress	0.00E + 00	0.00E + 00
	1	GO:0055114	Oxidation-reduction process	4.06E-09	8.58E-07
	1	GO:0006457	Protein folding	1.86E-06	3.92E-04
	1	GO:0006811	Ion transport	2.56E-06	5.40E-04
	1	GO:0009439	Cyanate metabolic process	1.35E-04	2.85E-02
	15	GO:0006950	Response to stress	1.12E-11	6.52E-09
	15	GO:0043335	Protein unfolding	4.16E-10	2.42E-07
	15	GO:0033383	Geranyl diphosphate metabolic process	1.80E-09	1.05E-06
	15	GO:0046248	Alpha-pinene biosynthetic process	6.42E-07	3.73E-04
	15	GO:0034605	Cellular response to heat	9.16E-07	5.32E-04
	15	GO:0043693	Monoterpene biosynthetic process	2.85E-06	1.66E-03
	15	GO:0045493	Xylan catabolic process	4.07E-05	2.37E-02

R: resistant phenotype; S: susceptible phenotype; I: PWN inoculation; W: water inoculation.

**Table 2:** DEGs involved in the biological process of syncytium formation (GO:0006949) in the resistant phenotype.

Dpi	ID	Annotation	Regulated
1	c69842.graph_c0	Pectate lyase precursor	2.54
	c72778.graph_c0	Expansin	1.40
	c81022.graph_c0	Expansin	5.48
	c72881.graph_c0	Expansin	1.82
15	c68276.graph_c0	Expansin	3.24
	c72881.graph_c0	Expansin	1.85
	c49518.graph_c0	Protein RALF-like 34	5.08

### Characterization of gene expression related to syncytium formation

**Figure 3:**
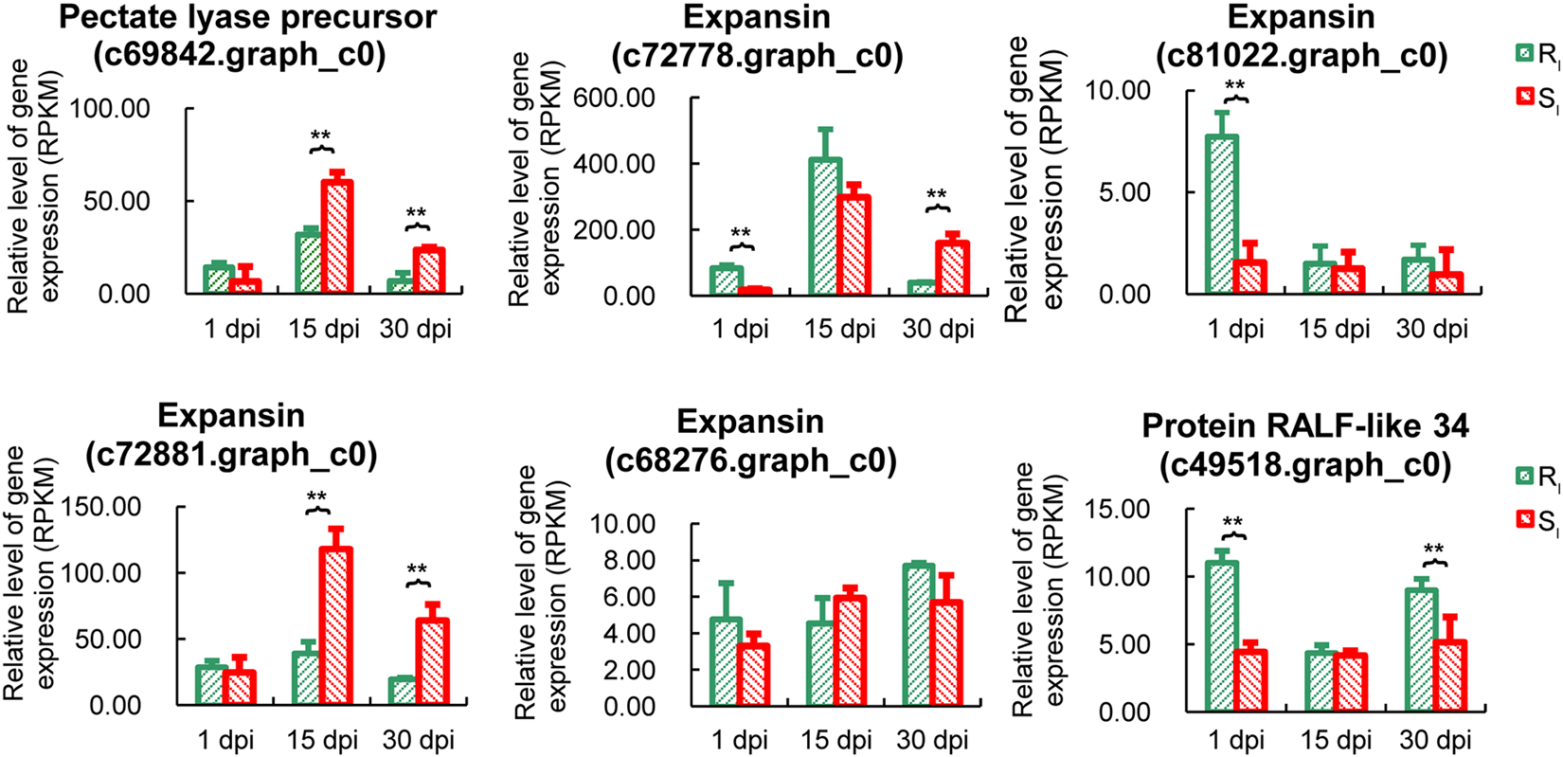
Difference in expression of six DEGs between resistant and susceptible phenotypes after PWN inoculation. These DEGs are involved in the biological process of syncytium formation in the resistant phenotype. *0.01 < *P* < 0.05 and **P < 0.01. Error bars represent the SE.

To better understand whether the GO process “syncytium formation” contributed to PWN responses, we identified related genes based on the functional annotation. A total of 7 DEGs involved in the GO term “syncytium formation” were identified in the resistant phenotype at 1 dpi and at 15 dpi. At 1 dpi, three up-regulated DEGs encoding expansin (c72778.graph_c0, c81022.graph_c0 and c72881.graph_c0) and one up-regulated DEG encoding pectate lyase precursor (c69842.graph_c0) were found in the resistant phenotype (Table 2). In the susceptible phenotype, these unigenes were not found to be significantly different between inoculation with PWN or water and were down-regulated after inoculation with PWN. Simultaneously, the DEGs encoding two expansins (c72778.graph_c0 and c81022.graph_c0) had higher expression in the resistant phenotype than in the susceptible phenotype after inoculation with PWN at 1 dpi (Fig. 3).

At 15 dpi, two up-regulated DEGs encoding expansins (c68276.graph_c0 and c72881.graph_c0) and one up-regulated DEG encoding RALF-like protein (c49518.graph_c0) were found in the resistant phenotype (Table 2). In the susceptible phenotype, the expression level of these unigenes was not found to be significantly different between inoculation with PWN or water. Among these unigenes, the unigene encoding expansin (c72881.graph_c0) had a higher expression level in the susceptible phenotype than in the resistant phenotype after inoculation with PWN at 15 dpi (Fig. 3). The results indicated that the expression of expansin was required to reach certain thresholds to ensure syncytium formation and PWN invasion.

### Regulation of the terpenoid biosynthesis pathway contributed to PWN resistance

**Figure 4:**
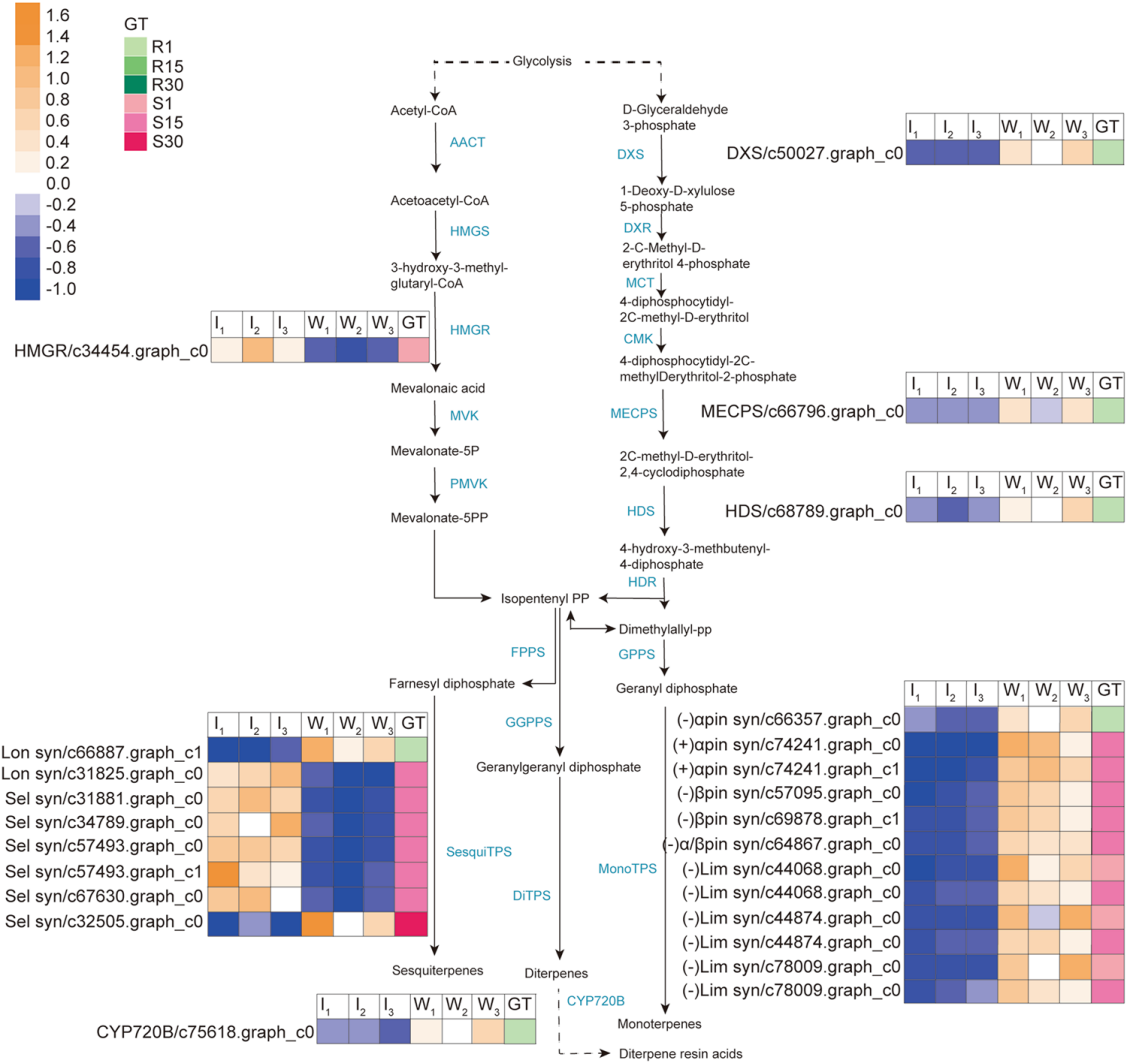
DEGs involved in terpenoid biosynthesis in stem of masson pine. **I** represents PWN inoculation, and **W** represents water control. The numbers following I and W represent the biological replicate. **GT** represents phenotype and time point. Enzymes involved in each step are shown in blue. DXS: 1-deoxy-D-xylulose-5-phosphate synthase; DXR: 1-deoxy-D-xylulose-5-phosphate reductoisomerase; MCT: 4-diphosphocytidyl-2C-methyl-D-erythritol synthase; CMK: 4-diphosphocytidyl-2C–methyl-D-erythritol kinase; MECPS: 2C-methyl-D-erythritol 4-phosphate cytidylyltransferase; HDS: 1-hydroxy-2-methyl-2-(E)-butenyl-4-diphosphate synthase; HDR: 1-hydroxy-2-methyl-2-(E)-butenyl-4-diphosphate reductase; AACT: acetoacetyl-CoA thiolase; HMGS: 3-hydroxy-3-methylglutaryl-CoA synthase; HMGR: 3-hydroxy-3-methylglutaryl-CoA reductase; MVK: mevalonate kinase; PMVK: phosphomevalonate kinase; MVD: mevalonate diphosphate decarboxylase; IPPI: isopentenyl-diphosphate isomerase; FPPS: farnesyl diphosphate synthase; GPPS: geranyl diphosphate synthase; SesquiTPS: sesquiterpene synthase; MonoTPS: monoterpene synthase; DiTPS: diterpene synthase; CYP720B: abietadienol/abietadienal oxidase PtAO.

**Figure 5:**
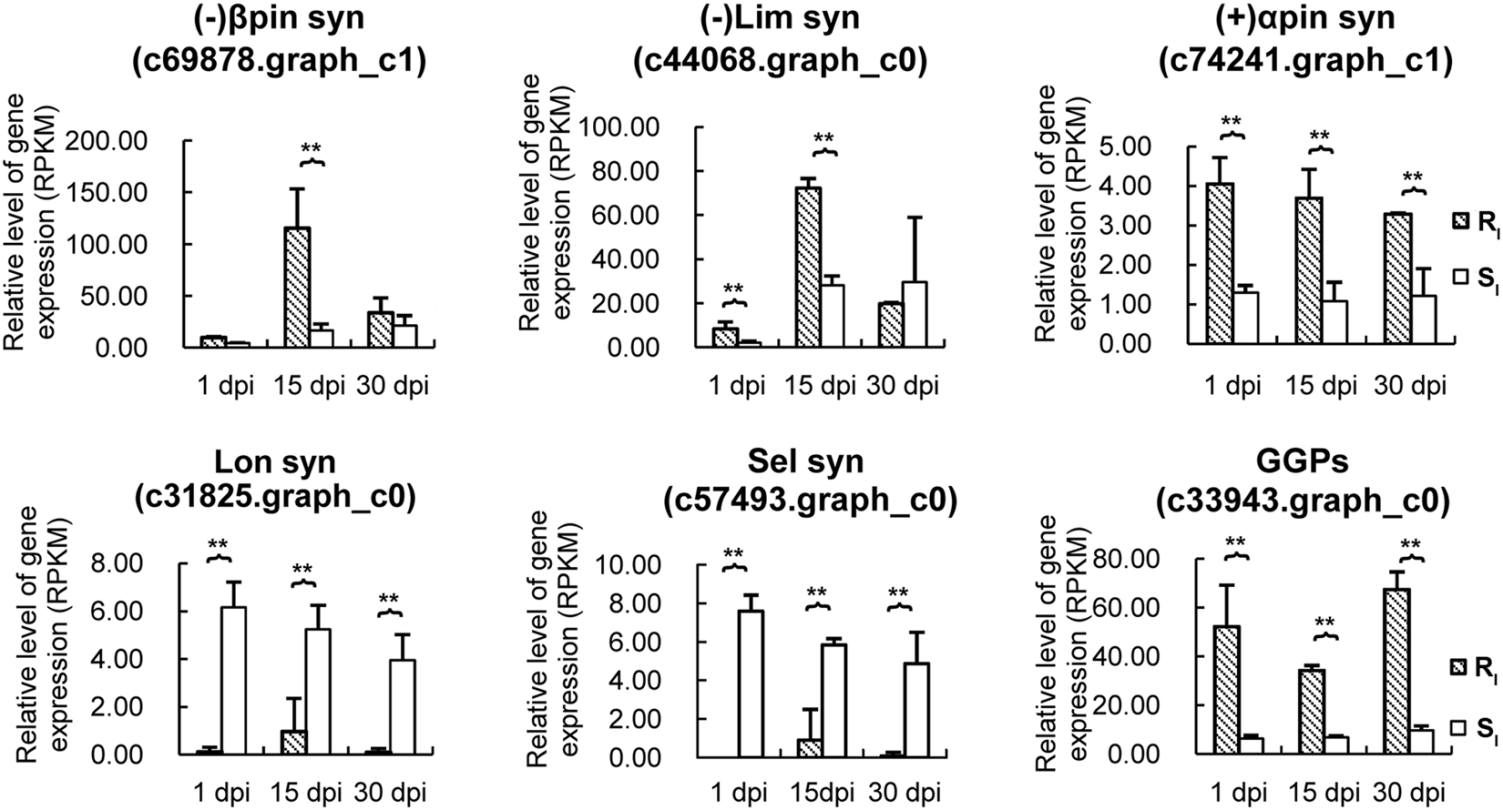
Difference in expression of terpene synthases and GGPS involved in terpenoid biosynthesis between resistant and susceptible phenotypes after PWN inoculation. Expression of these terpene synthases was significantly changed in resistant or susceptible phenotypes after PWN inoculation. Error bars represent SE. (−)βpin syn: (−)-β-pinene synthase; (−)Lim syn: (−)-Limonene synthase; (+)αpin syn: (+)-α-pinene synthase; Lon syn: longifolene synthase; Selsyn: delta-selinene synthase; GPPS: geranyl diphosphate synthase. Error bars represent the SE.

Terpenoids have been reported to play an important role in insect and pathogen resistance^26^. In this transcriptomics study, 21 DEGs involved in the biosynthesis of the terpenoid backbone were found in the two phenotypes (Fig. 4). Among these DEGs, the expression levels of 1-*D*-xylulose-5-phosphate synthese (DXS), 1-hydroxy-2-methyl-2-(*E*)-butenyl-4-diphosphate synthase (HDS), 2*C*-methyl-*D*-erythritol 4-phosphate cytidylyltransferase (MECPS), (−)-alpha-pinene synthase, longifolene synthase (c66887.graph_c1) and abietadienol/abietadienal oxidase PtAO (CYP720B) were down-regulated in the resistant phenotype at 1 dpi (Fig. 4). The expression of 3-hydroxy-3-methylglutaryl-CoA reductase (HMGR) and (−)-limonene synthase was up-regulated in the susceptible phenotype at 1 dpi (Fig. 4). Among these DEGs, the expression level of (−)-limonene synthase (c44068.graph_c0 and c78009.graph_c0) was significantly different between the resistant phenotype and the susceptible phenotype at 1 dpi (Fig. 5, Supplementary Fig. S3).

At 15 dpi, the DEGs encoding monoterpene synthases, including (−)-beta-pinene synthase, (+)-alpha-pinene synthase, (−)-limonene synthase and (−)-alpha/beta-pinene synthase, were down-regulated, and the DEGs encoding sesquiterpene synthases (longifolene synthase and delta-selinene synthase) were up-regulated in the susceptible phenotype (Fig. 4). Among these DEGs, the expression levels of (−)-beta-pinene synthase, (+)-alpha-pinene synthase, and (−)-limonene synthase (c44068.graph_c0 and c78009.graph_c0) were much higher in the resistant phenotype than in the susceptible phenotype after PWN inoculation at 15 dpi, but longifolene synthase (c31825.graph_c0) and delta-selinene synthase (c57493.graph_c0 and c57493.graph_c1) were expressed at higher levels in the susceptible phenotype (Fig. 5, Supplementary Fig. S3).

At 30 dpi, only delta-selinene synthase (c32505.graph_c0) was found to be down-regulated in the susceptible phenotype (Fig. 4). However, the expression level of the unigene was not significantly different between the resistant phenotype and the susceptible phenotype after PWN inoculation at 30 dpi.

In addition, three unigenes encoding geranylgeranyl pyrophosphate synthase (GGPPS) (c58549.graph_c0, c33360.graph_c0 and c33943.graph_c0) have higher expression in the resistant phenotype at 1, 15 and 30 dpi (Fig. 5, Supplementary Fig. S3), although the expression levels of these unigenes did not change significantly in the resistant phenotype or the susceptible phenotype after inoculation with PWN at every time point.

### Stress responsive pathways were involved in the resistance to PWN

We found that a total of 38 and 55 DEGs were involved in the significantly enriched GO term “response to stress” in the susceptible phenotype at 1 dpi and 15 dpi, respectively (Table 3). At 1 dpi, 37 down-regulated unigenes and one up-regulated unigene were found. Among the 37 down-regulated unigenes, there were 22 well characterized Hsps (i.e., 9 Hsp70, 4 Hsp 82, 4Hsp ST1, 2 Hsp 17.8, 1 Hsp 80, 1Hsp 83 and 1 Hsp90), 2 heat shock cognate proteins and 6 chaperone proteins. The only up-regulated unigene encoded a heat shock cognate protein (Supplementary Table S7). At 15 dpi, 52 down-regulated DEGs and 3 up-regulated DEGs were found. Among the 52 down-regulated DEGs, most of the DEGs encoded HSP and chaperone proteins. Simultaneously, there was one down-regulated unigene of ethylene-responsive transcription factor (ERF), which was also involved in the biological process of signal transduction and response to hormone. The up-regulated unigenes included 1 Hsp70 and 2 unknown proteins (Supplementary Table S7). Among the unigenes involved in the GO term “response to stress” in the susceptible phenotype at 1 dpi and 15 dpi, only one unigene encoding Hsp70 (c70394.graph_c0) was down-regulated significantly in the resistant phenotype at 15 dpi. These data highly suggested that substantial changes in the stress defense pathways were activated by PWN in the susceptible phenotype, while the resistant phenotype was more tolerate to PWN inoculation.

**Table 3:** DEGs involved in the GO term “response to stress” in the susceptible phenotype at 1 dpi and 15 dpi.

DEGs	1 dpi		15 dpi	
	up	down	up	down
HSP	0	22	1	23
Chaperone protein	0	7	0	11
Heat shock cognate protein	1	1	0	0
Hypothetical protein	0	4	0	4
Classical arabinogalactan protein	0	1	0	0
DnaJ protein homolog	0	1	0	2
Vegetative cell wall protein gp	0	1	0	0
ERF	0	0	0	1
Glycine-rich cell wall structural protein	0	0	0	1
Proline-rich receptor-like protein kinase	0	0	0	1
Molecular chaperone and allergen Mod-E/Hsp90/Hsp1	0	0	0	1
Mulatexin	0	0	0	1
Peroxidase	0	0	0	1
Probable mediator of RNA polymerase II transcription	0	0	0	4
Salicylate O-methyltransferase	0	0	0	1
Unknown	0	0	2	1

### The ROS scavenging pathway was required for PWN resistance

**Figure 6:**
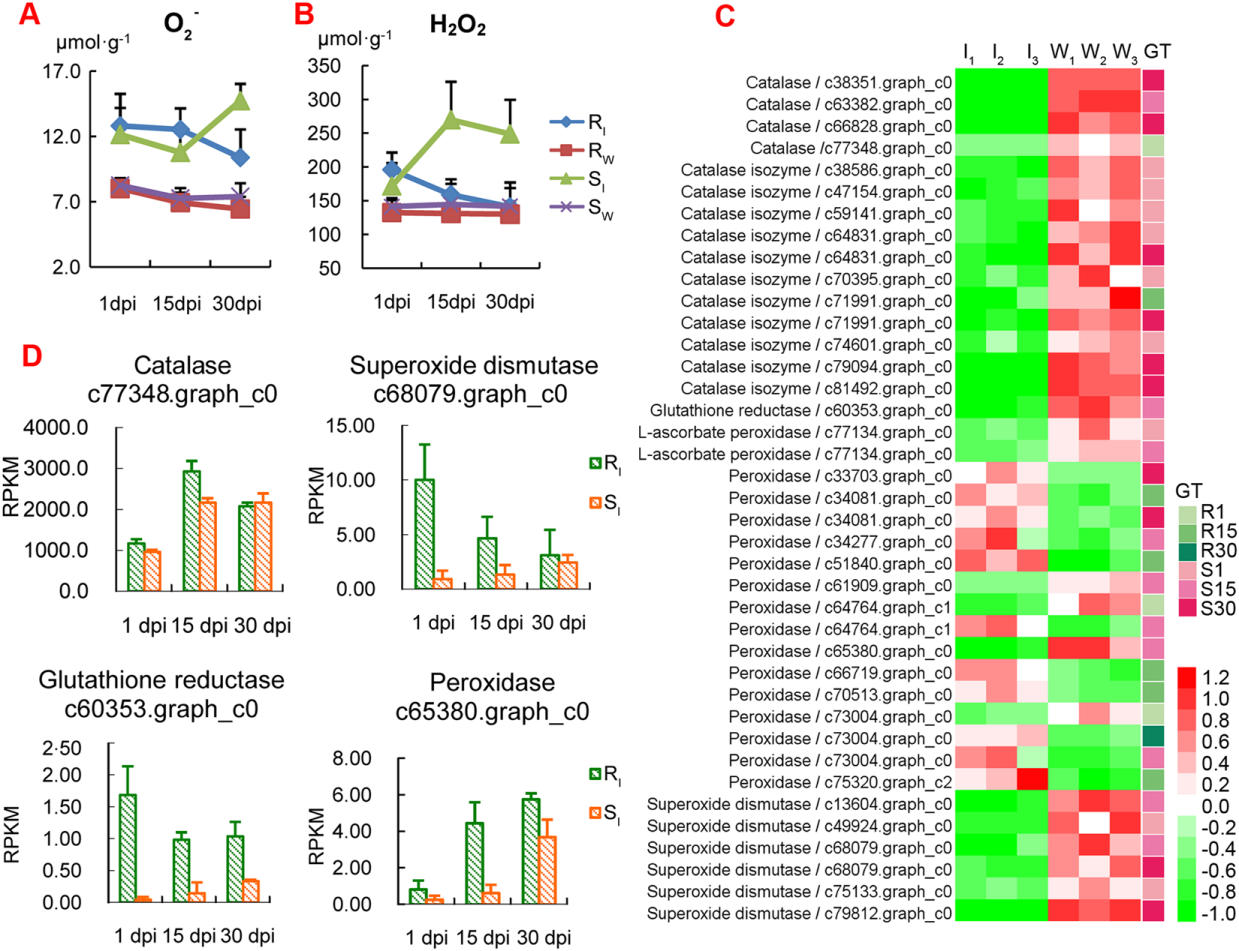
The content of ROS and the expression of genes encoding ROS-scavenging enzymes in resistant and susceptible phenotypes at three points. (**A**) Difference in O_2_^−^ content among resistant and susceptible phenotypes inoculating PWN and water. The results of multiple comparisons among resistant and susceptible phenotypes inoculated with PWN or water at the same time point are shown with lowercase letters. (**B**) Difference in H_2_O_2_ content among resistant and susceptible phenotypes inoculated with PWN and water. (**C**) Cluster analysis of DEGs encoding ROS-scavenging enzymes. The number following I and W represents the biological replicate. (**D**) Difference in expression of unigenes encoding catalase, superoxide dismutase, glutathione reductase and peroxidase between resistant and susceptible phenotypes after PWN inoculation. *0.01 < *P* < 0.05 and **P < 0.01. Error bars represent the SE.

To understand the physiological changes involved in PWN infection, we investigated the accumulation of ROS in PWN-inoculated masson pines. We showed that in both phenotypes, the concentration of superoxide radical (O_2_^−^) was significantly increased after inoculation of PWN at 1, 15 and 30 dpi (Fig. 6A). Furthermore, the concentration of O_2_^−^ was higher in the susceptible phenotype than in the resistant phenotype when inoculating PWN at 30 dpi. Although the concentration of hydrogen peroxide (H_2_O_2_) was significantly increased in the resistant phenotype at 1 dpi, it decreased gradually, and no significant difference was found between trees inoculated with PWN at 15 and 30 dpi. However, in the susceptible phenotype, the concentration of H_2_O_2_ increased quickly after inoculating with PWN, and it was much higher than in the control inoculated with water and the resistant phenotype inoculated with PWN (Fig. 6B). The results of the physiological experiment showed that more O_2_^−^ and H_2_O_2_ accumulated in the susceptible phenotype after inoculation with PWN.

From the transcriptome sequencing, a total of 33 DEGs related to ROS scavenging were identified in two phenotypes at three time points (Fig. 6C). Nine down-regulated DEGs encoding superoxide dismutase, glutathione reductase and L-ascorbate peroxidase were only found in the susceptible phenotype. Fifteen down-regulated DEGs encoding catalase or catalase isozyme were expressed in both phenotypes. The DEGs encoding peroxidase were down-regulated at 1 dpi and up-regulated at 15 and 30 dpi in the resistant phenotype, but no obvious trend was found in the susceptible phenotype. Among these DEGs, the expression levels of catalase (c77348.graph_c0), superoxide dismutase (c68079.graph_c0), glutathione reductase (c60353.graph_c0) and peroxidase (c65380.graph_c0) were involved in the biological process of defense response to nematode according to the annotation by the GO database (Fig. 6D), almost remaining higher in the resistant phenotype than in the susceptible phenotype at the three time points. These results suggested that the resistant phenotype was more competent to scavenge ROS, which was in good agreement with the physiological result.

#### Validation of RNA-seq expression data by qRT-PCR

**Figure 7:**
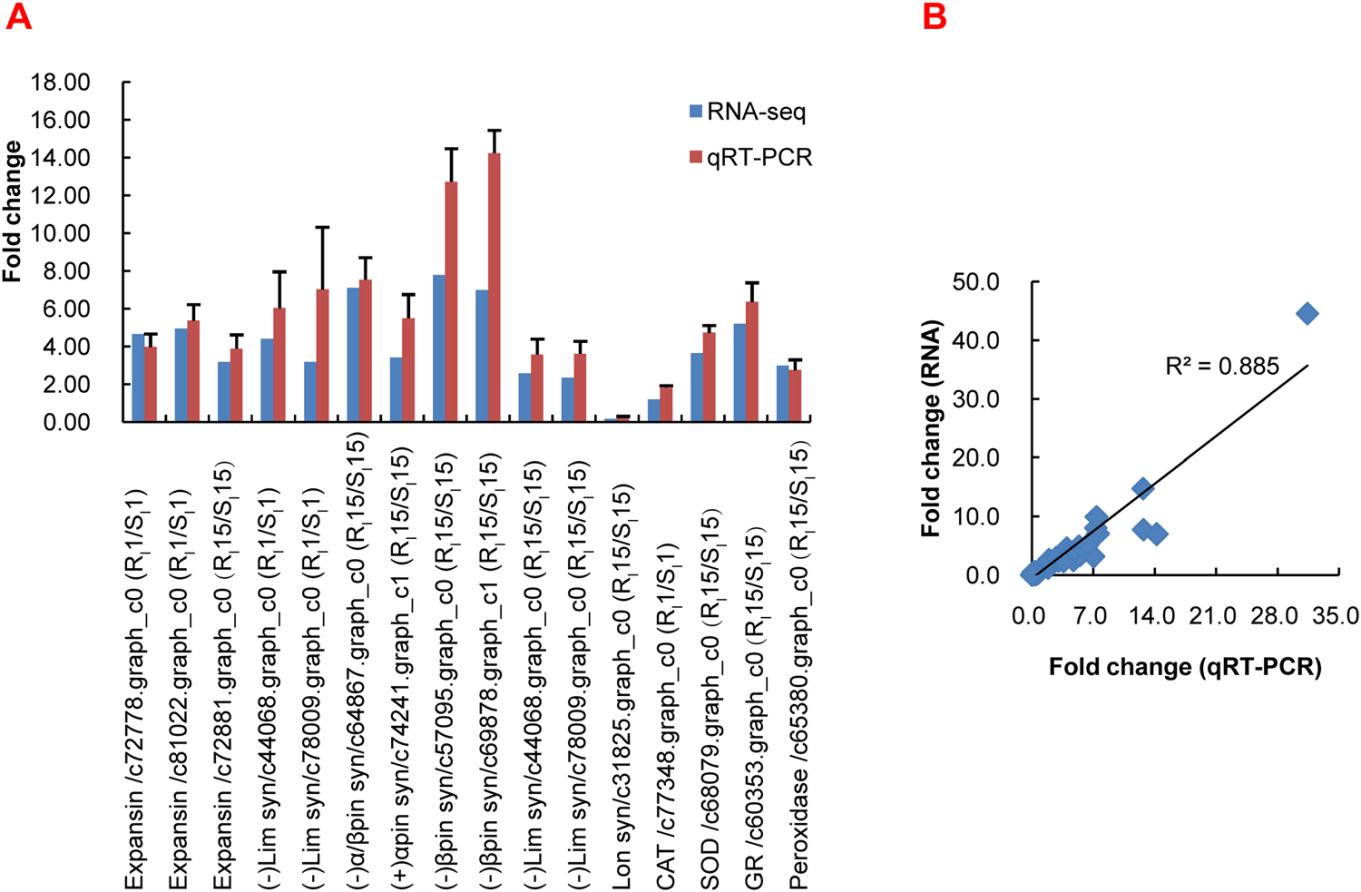
qRT-PCR validation of differentially expressed genes. (**A**) qRT-PCR validation of unigenes associated with resistance to PWN. (**B**) Correlation of 43 unigene expression results obtained from qPCR and RNA-seq. Relative expression levels of qRT-PCR calculated using Elongation factor 1-alpha as the internal control. The data are expressed as the mean (±SE) of three replicates. The expression data are presented as expression values of genes in the stigma sample relative to their expression. Error bars represent the SE.

To validate the reliability of the RNA-seq results, 43 unigenes from the DEGs described above were selected for qRT-PCR analysis (Supplementary Table S8). The expression of these unigenes was significantly different between the inoculated PWN and water trees for each phenotype or between the resistant and susceptible phenotype after PWN infection at 1, 15 or 30 dpi (Fig. 7A). The unigenes selected for qRT-PCR analysis were those involved in syncytium formation, terpenoid biosynthesis, pathogenesis-related genes, cell wall-related genes, heat shock protein and ROS responsive genes. The expression pattern of almost all unigenes indicated by qRT-PCR agreed well with RNA-seq except for one unigene (c68240.graph_c0). Simultaneously, a solid correlation was observed between the data for these unigenes from qRT-PCR and RNA-seq, with a correlation coefficient of 0.88 (Fig. 7B).

## Discussion

More DEGs were obtained between the resistant and susceptible masson pines using next-generation sequencing in our study than in Japanese black pines using suppression subtractive hybridization^22^. To identify high confidence DEGs in response to PWN, it is important to set appropriate controls for comparison in large-scale gene expression analysis. In this study, the control (inoculation with water) was set at each sampled time point in both resistant and susceptible masson pines, which enhanced the reliability of the obtained DEGs. In the resistant phenotype, the number of DEGs was always less than for the susceptible phenotype at each time point, which implied that the resistant masson pine is not easily interfered with by PWN compared with the susceptible masson pine. It has been shown that the resistance and susceptibility of plant species depend upon qualitative and quantitative differences in the activated genes in response to insects and pathogens^27^. According to the GO classification of DEGs, the difference in the significant GO terms in the resistant phenotype and susceptible phenotype indicated a qualitative difference in activated defense genes between the two phenotypes to PWN infection.

We observed that the most significant GO terms were “syncytium formation” for the DEG between inoculation with PWN or water in the resistant phenotype at 1 dpi and 15 dpi (Table 1). Syncytium formation is considered to be evoked by the nematode releasing secretions into initial syncytial cells; then, the partial cell wall between the initial syncytial cells and neighboring cells is dissolved, and the protoplasts are fused^28^. In this study, the DEGs involved in the biological process of syncytium formation included putative expansin, pectate lyase precursor and RALF-like protein. Expansins, which are cell wall-loosening proteins, were involved in the growth regulation of various cell types in response to different stimuli at different plant developmental stages^29^. In tomato and soybean, expansin was identified after inoculation with cyst nematodes^30, 31^, indicating the importance of cell wall modification during the plant defense response. Hirao *et al*.^22^ also reported that cell wall-related genes played a role in the effective defense response against PWN infection in *P. thunbergii*. RALF-like proteins, forming a large family, are small polypeptides^32^. Pearce *et al*.^33^ found that RALF was involved in the cell-cell signaling biological process and can activate intracellular MAPKs. Although previous studies have thus far revealed that expansins participated in cell wall disassembly and cell growth during syncytium formation when plants were infected with root-knot nematodes in roots^34^, syncytium was not found in the stem of masson pine after inoculation with PWN. However, it is well known that new traumatic resin ducts are induced in conifers under biotic or abiotic stress^35^, which are differentiated by clusters of swollen and irregularly shaped cells in the cambial zone. Nagy *et al*.^36^ also indicated that resistant trees induced traumatic resin ducts earlier than susceptible trees in response to fungal infection. During the development of traumatic resin ducts, the cell wall was changed greatly. Therefore, the function of expansin might be associated with the formation of traumatic resin ducts. In our study, we found that the expression of expansin was higher in resistant trees than susceptible trees at 1 dpi, but expansin was induced much more in susceptible trees at 15 dpi.

Oleoresin, a mixture of monoterpenes, sesquiterpenes and diterpene resin acids, plays an important role in defense against pathogens and insects in conifers^37, 38^. The biosynthesis of the terpenoid backbone is usually completed through two separate pathways of methyl-erythritol 4-phosphate (MEP) and mevalonate (MVA) in conifers. Monoterpenes and diterpenes are biosynthesized by the MEP pathway^39^, in which the enzymes of DXS, 1-deoxy-*D*-xylulose-5-phosphate reductoisomerase (DXR), 4-diphosphocytidyl-2*C*-methyl-*D*-erythritol synthase (MCT), 4-diphosphocytidyl-2*C*-methyl-*D*-erythritol kinase (CMK), MECPS, HDS, 1-hydroxy-2-methyl-2-(*E*)-butenyl-4-diphosphate reductase (HDR), geranyl diphosphate synthase (GPPS), mono-TPS, di-TPS and cytochrome P450 (CYP450)-dependent monooxygenase are involved. HDS was also involved in the processes of the negative regulation of defense response and plant-type hypersensitive response according to GO annotation. The biosynthesis of sesquiterpenes is completed by the MVA pathway^39^, in which the enzymes of acetoacetyl-CoA thiolase (AACT), 3-hydroxy-3-methylglutaryl-CoA synthase (HMGS), HMGR, mevalonate kinase (MVK), phosphomevalonate kinase (PMVK), mevalonate diphosphate decarboxylase (MVD), farnesyl diphosphate synthase (FPPS) and Sesqui-TPS are involved. CYP450 can mediate the oxidative reactions involved in the biosynthesis of both primary and secondary metabolites in plants. To date, only two P450 genes, CYP720B1 from loblolly pine (*P. taeda*) and CYP720B4 from white spruce (*Picea sitchensis*), which take part in the formation of the diterpene resin acids of oleoresin, have been functionally characterized^40, 41^.

After the trunk of a conifer suffers an insect attack, pathogen invasion or mechanical wounding, inducible oleoresin can be synthesized in addition to constitutive oleoresin, and the volume and composition of oleoresin varies depending by herbivores and pathogens^36, 42, 43^. The turpentine (monoterpenes and sesquiterpenes) can directly affect herbivores through emitting toxic volatiles, and diterpene resin acids present physical barriers at the wound^38, 44, 45^. Among the components of oleoresin, limonene is considered the most abundant toxic substance to beetles^46^. Zhao *et al*.^47^ reported that the concentration of longifolene was changed after inoculation with PWN in masson pine. As the products of (+)- and (−)-alpha-pinene synthase, (+)-alpha-pinene and (−)-alpha-pinene present simultaneously in masson pine. Alpha-pinene is a mixture of the isomers (+)-alpha-pinene and (−)-alpha-pinene. The content of (−)-alpha-pinene is approximately 10- to 20-fold higher than (+)-alpha-pinene in masson pines^41^. The mono-TPS and sesqui-TPS might play important roles in controlling PWN infestation. The candidate genes of (−)-beta-pinene synthase, (+)-alpha-pinene synthase, (−)-alpha/beta-pinene synthase, and (−)-limonene synthase might play positive regulatory roles, and the candidate genes of longifolene synthase and delta-selinene synthase might play negative regulatory roles. In this study, we found that the expression levels of many genes involved in terpenoid biosynthesis were changed after PWN inoculation. However, among these unigenes, the expressed difference of DXS, HDS, MECPS, HMGR, and (−)-alpha-pinene synthase was not significant after PWN inoculation between the resistant phenotype and the susceptible phenotype. The arrangement of the resin ducts might affect PWN activities. Kuroda^48^ reported that intricate and winding structure of resin ducts at the joints between the branch and the main stem might be effective in retarding PWN. In *P. massoniana*, we found that the resistant phenotype had a larger resin duct size, area and number than the susceptible phenotype when the trees were in the original state (Fig. 1A). The area of the axial resin canals was positively related to the oleoresin yield in *P. pinaster* ^49^. The trees having a large amount of oleoresin had stronger resistance in loblolly pine^50^. In this study, although the relationship between GGPPS and the defense response to PWN infestation is not clear, it is interesting that the expression of GGPPS has a higher level in resistant than in susceptible trees. Our previous study demonstrated that GGPPS was expressed at higher levels in the high oleoresin-yielding phenotype than in the low oleoresin-yielding phenotype of masson pine^51^. Therefore, resistant masson pines might have a higher oleoresin yield than susceptible ones. However, except for the genes encoding CYP450, no genes involved in terpenoid biosynthesis had differential expression between resistant and susceptible trees in *P. thunbergii* ^22^, which suggested that the defense mechanism against PWN may vary by tree species.

In addition to oleoresin, releasing ROS as signal molecules or toxic molecules plays an important role in pathogen defense^52^. Hirao *et al*.^22^ found that the genes involved in the ROS scavenging were rapidly induced in *P. thunbergii* infected with PWN. The major ROS-scavenging enzymes of plants include superoxide dismutase, ascorbate peroxidase, catalase, monodehydroascorbate, dehydroascorbate reductase, glutathione reductase, and glutathione peroxidase^53^. As the H_2_O_2_ scavenging enzyme, ascorbate peroxidase might be responsible for the fine modulation of ROS for signaling purposes, but catalase might be responsible for removing excess ROS during stress. In this study, we found that the content of ROS (O_2_^−^ and H_2_O_2_) was increased after inoculating PWN in both the resistant and susceptible phenotypes at 1 dpi, and the content of the ROS in the resistant phenotype then decreased gradually at 15 and 30 dpi (Fig. 6A,B). Especially for H_2_O_2_, the content was almost maintained at the normal level in the resistant phenotype at 30 dpi. From the results of the transcriptome, the expression levels of ROS scavengers, such as catalase, superoxide dismutase, peroxidase and glutathione reductase, were higher in the resistant phenotype than in the susceptible phenotype at almost every time point, which indicated that the resistant phenotype had a stronger ROS-scavenging capability. The enhanced H_2_O_2_ can induce the expression of PRs and phytoalexin in plants^54^. PR-1, PR-2 and PR-5 are salicylic acid-responsive genes, and PR-6 is a jasmonic acid and ethylene responsive gene^55^. In our study, the expression of PR-1, PR-3, PR-5, PR-9 and PR-10 was changed significantly in the resistant or susceptible phenotype after PWN inoculation. However, no obvious trend was observed for PR-1, PR-3, PR-5 and PR-10. Therefore, PRs (PR-1, PR-3, PR-5 and PR-10) might not be effective in controlling PWN infection, which is consistent with the result for *P. thunbergii* ^22^.

## Conclusion

The gene expression profiling between resistant and susceptible masson pines after inoculation with PWN is studied for the first time in this work. We systematically identified genes that were potentially related to PWN resistance using a combination of DEG analyses. We showed that most of the DEGs were down-regulated, possibly leading to the physiologic disorders in the susceptible phenotype. We revealed that the resistant and susceptible phenotypes had a different defense mechanism in response to PWN. The GO processes of “syncytium formation,” enriched in the resistant phenotype, and “response to stress,” enriched in the susceptible phenotype, highlighted the different roles of the underlying pathways that were related to PWN tolerance. Moreover, detailed gene expression analysis suggested that terpenoids were prominent defense compounds against PWN. The mono-TPS of (−)-beta-pinene synthase, (+)-alpha-pinene synthase, (−)-alpha/beta-pinene synthase, and (−)-limonene synthase might play positive regulatory roles, and the sesqui-TPS of longifolene synthase and delta-selinene synthase might play negative regulatory roles in resistance against PWN infection. In addition, the higher activity of ROS-scavenging enzymes was effective to inhibit the death of resistant masson pines after PWN inoculation. This study provides a starting point for understanding a successful defense mechanism against PWN in masson pine. In future studies, more detailed functional analysis of thekey genes obtained in this study should be carried out to unravel the complex defense mechanisms.

## Materials and Methods

### Plant materials

#### Plant materials and treatments

A resistant variety of masson pine, ‘Xiuning 5,’ which has the higher resistance to PWN (mortality rates of its clones were all 0% after inoculating with PWN at three sites in the previous test) and is selected from 324 resistant families, was planted in the germplasm nursery of Anhui Academy of Forestry, Anhui Province, China. A susceptible variety ‘Huangshan 1’ (mortality rates of its clones were all 100% after inoculation with PWN at three sites in the previous test), selected as the control, was also planted in the germplasm nursery of the Anhui Academy of Forestry. Both clones were obtained from the original trees using the pith-cambium pairing grafting method onto 2-year-old seedlings at the Anhui Academy of Forestry in 2010. The PWN used in our study was the highly virulent Guangzhu-3B isolate.

The experiment was conducted using eighteen four-year-old ramets for each clone on July 20, 2014. The average heights of the resistant and susceptible clones were 2.43 m and 2.51 m, respectively. The tree branch approximately 2 cm below the node of the annual shoot was cut at a slant with a knife so that it was approximately 3 cm long and 1 mm deep into the xylem. Then, the section was scraped using a saw to imitate bites from cerambycid beetles. Subsequently, 10,000 nematodes suspended in 20 µL sterile water were injected into the longitudinal wound of nine ramets of each clone. For the other nine ramets of each clone, only sterile water was injected into the wound as a control. At 1, 15 and 30 dpi, the stem tissue of inoculated PWN trees and control trees was collected 5 cm above the inoculation point. Three ramets for each clone were selected as three biological replicates. Then, a 2 cm-long segment of stem was cut off and put into liquid nitrogen immediately in the field. Finally, these samples were stored at −80 °C for RNA extraction. Simultaneously, the needles per sample above the 1 cm inoculation point were also taken and stored at −20 °C for ROS measurement. The last sampled time was according to previous results that the needles of susceptible trees turned yellow after 30 dpi, which was observed in this experiment.

#### Total RNA isolation and cDNA synthesis

Total RNA from each sample of inoculated PWN trees and control trees at 1, 15 and 30 dpi was separately extracted using the Plant RNA kit RN38 EASYSpin plus (Aidlab Biotech, Beijing, China), containing DNA digestive enzyme. The concentration of the total RNA was detected using an Ultraspec TM 2100 Pro UV/visible spectrophotometer, and the integrity of the total RNA was detected using 1% agarose gel electrophoresis. Then, the integrity of the RNA was also tested using an Agilent 2100 Bioanalyzer before proceeding. High-quality RNA was defined as having a concentration of more than 400 ng/μL, OD260/280 ranging from 1.8 to 2.2, OD260/230 over 2.0, 28S/18S no less than 1.5 and RIN over 8.0. A total of thirty-six RNA samples were obtained, including three biological replicates for inoculated PWN trees and control trees at three time points.

### Transcriptome sequencing and assembly of masson pine

To obtain a comprehensive list of transcripts, equal amounts of high-quality RNA from thirty-six RNA samples were pooled together. Then, the total RNA was delivered to the Biomarker Technology Company (Beijing, China) to construct a cDNA library and perform sequencing. The cDNA library was paired-end sequenced on the Illumina HiSeqTM 2000 sequencing platform, and 2× 101 bp reads were produced from either end of a cDNA fragment.

After filtering the raw reads, we assembled the high-quality reads into contigs using the Trinity method^56^. Then, the transcripts were assembled with the paired-end information of the contigs. Finally, the longest transcripts from the potential alternative splicing transcripts were selected as the unigenes.

### Differentially expressed genes and GO enrichment

All high-quality reads of 36 samples were aligned to the assembled transcripts above using Bowtie, allowing no more than one nucleotide mismatch. The data has been submitted to NCBI, and the accession number is SRP103562. To compare the expression abundance among 36 samples, RPKM was used to represent the expression abundance of the unigenes. The differential expression of genes was analyzed by the edgeR package (Version 3.2.4) in BioConductor (release 2.12, R version 2.15.0)^57^. The right-sided hypergeometric enrichment test was performed at a medium network specificity selection, and the p-value correction was performed using the Benjamini-Hochberg method. A GO enrichment analysis of the DEGs was performed using the GOseq R package, and GO terms with a corrected p-value below 0.05 were considered to have significant enrichment. Heatmaps and hierarchical clustering analysis were performed using the HemI software (Heatmap Illustrator, version 1.0.3.3)^58^, with the data normalized as in Equation (1):

1 $${y}_{ij}=\frac{{{\rm{x}}}_{ij}-u}{u}$$

where *y*_*ij*_ represents the value of the *j*th replicate of the *i*th phenotype used in the heatmap and hierarchical clustering (*i* = 1 to 2 and *j* = 1 to 3), *x*_*ij*_ represents the value of the *j*th replicate of the *i*th phenotype obtained by RNAseq, and *u* is the overall mean.

### Quantitative RT-PCR analysis

The RNA samples used in the qRT-PCR and transcriptome sequencing were identical. The primer pairs (Supplementary Table S9) for the representative candidate genes were designed using the Primer 3.0 software with the optimal Tm at 58–62 °C, primer length of 19–21 nucleotides, PCR product size of 120–200 bp and GC content of 45–55%.

Quantitative RT-PCR was run in a 7300 Real Time PCR System (Applied Biosystems, CA, USA) with the SYBR Green detection method to verify the transcriptome sequencing results. The cDNA was amplified in a total volume of 20 μL, including 0.4 μL of ROX reference dye, 2 μL of cDNA, 0.4 μL of primers and 10 μL of SYBR Premix ExTaqTM. The PCR program was 95 °C for 10 s and 40 cycles of 95 °C for 5 s, 58 °C for 30 s and 72 °C for 30 s. Three technical replicates for each of the three biological replicates were performed. The transcript profiles were normalized using the reference gene of elongation factor 1-alpha, and the relative expression levels of candidate genes were calculated with the 2^−ΔΔCt^ method^59^.

### Anatomical measurements

Approximately 1 cm stem segments were taken from thirty non-inoculated trees of resistant and susceptible phenotypes for anatomical observation. These stem segments were stored in 70% ethanol. Sections of each sample were obtained and stained with 1% safranine following standard protocols^60^. After dehydrating via an ethanol series, the sections were mounted. Samples were photographed using a Leica DM 4000B light microscope with a Leica DFC450 digital camera and analyzed with Leica Application Suite (version4.5). The parameters of the mean size of the axial canals (AC size), the area of the axial canals per mm^2^ (AC area) and the number of radial canals per mm^2^ (AC freq) in the cross section were calculated for each sample. The mean values of the resistant phenotype and susceptible phenotype were compared by *t*-test (α = 0.05).

### H_2_O_2_ and O_2_^−^ quantification

H_2_O_2_ was extracted from needles and measured according to Doulis’method^61^. O_2_^−^ was extracted and measured according to Liu’s hydroxylamine method^62^. At each time point, the significant difference in the O_2_^−^ content between the four treatments (two phenotypes inoculating PWN and water, respectively) was determinedbyLSD test (α = 0.05).

## Electronic supplementary material

Supplementary Information

Supplementary Table S4

Supplementary Table S7

## References

[1] Yano M (1913). Investigation on the cause of pine mortality in Nagasaki. Prefecture sanrinkoho..

[2] Cheng H, Lin M, Li W, Fang Z (1983). The occurrence of a pine wilting disease caused by a nematode found in Nanjing. For Pest Dis..

[3] Dwinell LD (1993). First report of pine wood nematodes (*Bursaphelenchus xylophilus*) in Mexico. Plant Dis..

[4] Mota MM, Braasch H, Bravo MA (1999). First record of *Bursaphelenchus xylophilus* in Portugal and in Europe. Nematology..

[5] Kobayashi F, Yamane A, Ikeda T (1984). The Japanese pine sawyer beetle as the vector of pine wilt disease. Annu Rev Entomol..

[6] Yang BJ (2002). Advanced in research of mechanism of pine wood nematode. Forest Pest and Disease..

[7] Sousa, E., Rodrigues, J. M., Bonifácio, L. F., Naves, P. M., & Rodrigues, A. Nematodes: Morphology, Functions and Management Strategies (eds Boeri, F. and Chung, J.A.) Ch. 6, 157–178 (Nova Science, 2011).

[8] Yang BJ, Liu W, Xu FY, Zhang P, Qu HR (1999). Study on early diagnosis for pine wilt disease caused by *Bursaphelenchus xylophilus*. II. The effect of pine species, dose and nematode origin on oleoresin exudation method. Forest Reseach..

[9] Toda T, Kurinobu S (2001). Genetic improvement in pine wilt disease resistance in *Pinus thunbergii*: The effectiveness of pre-screening with an artificial inoculation at the nursery. Journal of Forest Research..

[10] Xu LY (2012). Study on the disease resistance of candidate clones in *Pinus massonina* to *Bursaphelenchus xylophilus*. China Forestry Science and Technology..

[11] Ribeiro B (2012). Pine wilt disease: detection of the pinewood nematode (*Bursaphelenchus xylophilus*) as a tool for a pine breeding programme. Forest Pathology..

[12] Toda T (2004). Studies on the breeding for resistance to the pine wilt disease in *Pinus densiflora* and *P. thunbergii*. Bull Forest Tree Breed Center.

[13] Ahn SH, Jeon MJ, Eom YG, Oh SC, Lee MR (2011). Wood anatomical characteristics of domestic red pine (*Pinus densiflora*) infested by pine wood nematode (*Bursaphelenchus xylophilus*). Journal of the Korean Wood Science & Technology..

[14] Kuroda K (1989). Terpenoids causing tracheid-cavitation in *Pinus thunbergii* infected by the pine wood nematode (*Bursaphelenchus xylophilus*). Ann. Phytopath. Soc. Jpn..

[15] Ishida K, Hogetus T (1997). Role of resin canals in the early stage of pine wilt disease caused by the pine wood nematode. Can. J. Bot..

[16] Oku H, Shiraishi T, Kurozumi S (1979). Participation of toxin in wilting of Japanese pines caused by a nematode. Naturwissenschaften..

[17] Vicente, C. *et al*. Natural bacterial communities associated with the pine sawyer beetle *Monochmus galloprovincialis*. *Pine Wilt Disease Conference*. 63–64 (2013). 10.1111/1574-6968.12232 23927049

[18] Kikuchi T, Jones JT, Aikawa T, Kosaka H, Ogura N (2004). A family of glycosyl hydrolase family 45 cellulases from the pine wood nematode *Bursaphelenchus xylophilus*. FEBS Letters..

[19] Fukuda K (1997). Physiological process of the symptom development and resistance mechanism in the wilt disease. J. For. Res.

[20] Shin H (2009). Identification of genes upregulated by pinewood nematode inoculation in Japanese red pine. Tree Physiol..

[21] Nose M, Shiraishi S (2011). Comparison of the gene expression profiles of resistant and non-resistant Japanese black pine inoculated with pine wood nematode using a modified Long SAGE technology. For. Pathol..

[22] Hirao T, Fukatsu E, Watanabe A (2012). Characterization of resistance to pine wood nematode infection in *Pinus thunbergii* using suppression subtractive hybridization. BMC Plant Biology..

[23] Santos CSS, Vasconcelos MW (2011). Identification of genes differentially expressed in *Pinus pinaster* and *Pinus pinea* after infection with the pine wood nematode. Eur. J. Plant Pathol..

[24] Yang BJ (1987). The resistance of pine species to pine wood nematode *Bursaphelenchus xylophilus*. Acta Phytopathologica Sinica..

[25] Xu L (2013). Characterization of the *Pinus massoniana* transcriptional response to *Bursaphelenchus xylophilus* infection using suppression subtractive hybridization. Int. J. Mol. Sci..

[26] Keeling CJ, Bohlmann J (2006). Genes, enzymes and chemicals of terpenoid diversity in the constitutive and induced defense of conifers against insects and pathogens. New Phytol..

[27] Thompson GA, Goggin FL (2006). Transcriptomics and functional genomics of plant defense induction by phloem-feeding insects. J Exp Bot.

[28] Golinowski W, Grundler FMW, Sobczak M (1996). Changes in the structure of Arabidopsis thaliana during female development of plant-parasitic nematode *Heterodera schachtii*. Protoplasma..

[29] Li Y (2002). Plant expansins are a complex multigene family with an ancient evolutionary origin. Plant Physiol..

[30] Fudali S (2008). Two tomato α-expansins show distinct spatial and temporal expression patterns during development of nematode induced syncytia. Physiol Plant..

[31] Matsye PD (2011). Mapping cell fate decisions that occur during soybean defense responses. Plant Mol Biol..

[32] Olsen AN, Mundy J, Skriver K (2002). Peptomics, identification of novel cationic *Arabidopsis* peptides with conserved sequence motifs. In Silico Biol..

[33] Pearce, G., Moura, D. S., Stratmann, J. R. & Jr, C. A. RALF, a 5-kDa ubiquitous polypeptide in plants, arrests root growth and development. *Proceedings of the National Academy of Sciences*, USA **98**, 12843–12847 (2001). 10.1073/pnas.201416998 PMC60141 11675511

[34] Wieczorek K (2006). Expansins are involved in the formation of nematode-induced syncytia in roots of Arabidopsis thaliana. Plant Journal..

[35] Byun-McKay A (2006). Wound-induced terpene synthase gene expression in Sitka spruce that exhibit resistance or susceptibility to attack by the white pine weevil. Plant Physiol..

[36] Nagy N, Franceschi VR, Solheim H, Krekling T, Christiansen E (2000). Wound-induced traumatic resin duct development in stems of Norway spruce (*Pinaceae*): anatomy and cytochemical traits. Am. J. Bot..

[37] Trapp S, Croteau R (2001). Defensive resin biosynthesis in conifers. Annu Rev Plant Physiol Plant Mol Biol..

[38] Martin DM, Bohlmann J (2005). Molecular biochemistry and genomics of terpenoid defenses in conifers. Rec Adv Phytochem..

[39] Zulak KG, Bohlmann J (2010). Terpenoid biosynthesis and specialized vascular cells of conifer defense. J. Integr Plant Biol..

[40] Ro DK, Bohlmann J (2006). Diterpene resin acid biosynthesis in loblolly pine (*Pinus taeda*): functional characterization of abietadiene/levopimaradiene synthase (PtTPS-LAS) cDNA and subcellular targeting of PtTPS-LAS and abietadienol/abietadienal oxidase (PtAO, CYP720B1). Phytochemistry..

[41] Hamberger B, Ohnishi T, Hamberger B, Seguin A, Bohlmann J (2011). Evolution of diterpene metabolism: Sitka spruce CYP720B4 catalyses multiple oxidations in resin acid biosynthesis of conifer defense against insects. Plant Physiol..

[42] Clark EL, Huber DPW, Carroll AL (2012). The legacy of attack: implications of high phloem resin monoterpene levels in lodgepole pine following mass attack by mountain pine beetle, *Dendroctonus ponderosae* Hopkins. Environ. Entomol..

[43] Ott DS, Yanchuk AD, Huber DPW, Wallin KF (2011). Genetic variation of lodgepole pine, *Pinus contorta* var. *latifolia*, chemical and physical defenses that affect mountain pine beetle, *Dendroctonus ponderosae*, attack and tree mortality. J. Chem. Ecol..

[44] Miller B (2005). Insect-induced conifer defense: White pine weevil and methyl jasmonate induce traumatic resinosis, de novo formed volatile emissions, and accumulation of terpenoid synthase and putative octadecanoid pathway transcript in Sitka spruce. Plant Physiol..

[45] Bohlmann J (2012). Pine terpenoid defences in the mountain pine beetle epidemic and in other conifer pest interactions: specialized enemies are eating holes into a diverse, dynamic and durable defence system. Tree Physiology..

[46] Sturgeon KB (1979). Monoterpene variation in ponderosa pine xylem related to western pine predation. Evolution..

[47] Zhao ZD (2001). Study on chemical components and resistance mechanism to pine wood nematode of masson pine provenance. Chemistry and Industry of Forest Products..

[48] Kuroda K (2004). Inhibiting factors of symptom development in several Japanese red pine (*Pinus densiflora*) families selected as resistant to pine wilt. J For Res..

[49] Rodriguez-Garcia A, Lopez R, Martin JA, Pinillos F, Gil L (2014). Resin yield in *Pinus pinaster* is related to tree dendrometry, stand density and tapping-induced systemic changes in xylem anatomy. For. Ecol. Manage..

[50] Strom BL (2002). Oleoresin characteristics of progeny of loblolly pines that escaped attack by southern pine beetle. For. Ecol. Manage..

[51] Liu QH (2015). Genome-Wide identification of differentially expressed genes associated with the high yielding of oleoresin in secondary xylem of masson pine (*Pinus massoniana* Lamb.) by transcriptomic analysis. PloS ONE.

[52] Barcelo, A. R. & Laura, V. G. R. Reactive oxygen species in plant cell walls (*eds Del Rio LA and Puppo A*), 73–93 (*Sringer-Verlag Berlin Heidelberg, 2009*).

[53] Mittler R (2002). Oxidative stress, antioxidants, and stress tolerance. Trends Plant Sci J..

[54] Klessig DF, Malamy J (1994). The salicylic acid signal in plants. Plant Mol. Biol..

[55] Ohtsubo N, Mitsuhara I, Koga M, Seo S, Ohashi Y (1999). Ethylene promotes the necrotic lesion formation and basic PR gene expression in TMV-infected tobacco. Plant Cell Physiol..

[56] Haas BJ (2013). Denovo transcript sequence reconstruction from RNA-Seq: Trinity platform for reference generation and analysis. Nat Protoc..

[57] Robinson MD, McCarthy DJ, Smyth GK (2010). edge R: a Bioconductor package for differential expression analysis of digital gene expression data. Bioinformatics..

[58] Deng WK, Wang Y, Liu Z, Cheng H, Xue Y (2014). HemI: A Toolkit for Illustrating Heatmaps. Plos One.

[59] Livak KJ, Schmittgen TD (2001). Analysis of relative gene expression data using real-time quantitative PCR and the 2^−ΔΔCt^ method. Methods..

[60] Heijari J (2005). Application of methyl jasmonate reduces growth but increases chemical defence and resistance against *Hylobius abietis* in Scots pine seedlings. Entomol. Exp. Appl..

[61] Doulis AG, Debian N, Kingstonsmith AH, Foyer CH (1997). Differential localization of antioxidants in maize leaves. Plant Physiol..

[62] Liu J, Wu XQ, Ying CX, He LX, Ye JR (2009). The difference of progenitive power and superoxide anion production in *Bursaphelenchus xylophilus* and *B. mucronatus*. Journal of Nanjing Forestry University.

