# Supplementary material for: Transcriptomic Profiling Reveals Differentially Expressed Genes Associated with Pine Wood Nematode Resistance in Masson Pine (Pinus massoniana Lamb.)
Source: Sci Rep. 2017 Jul 5;7:4693. doi: 10.1038/s41598-017-04944-7 (PMC5498564; doi:10.1038/s41598-017-04944-7)
Supplement: Supplementary file 1 — Supplementary Information [file 41598_2017_4944_MOESM1_ESM.doc]

**Transcriptomic Profiling Reveals Differentially Expressed Genes Associated with Pine Wood Nematode Resistance in Masson Pine (*Pinus massoniana* Lamb.)**

Qinghua Liu^1,2^, Yongcheng Wei^1,2^, Liuyi Xu^3^, Yanping Hao^3^, Xuelian Chen^3^, and Zhichun Zhou^1,2^*

^1^Research Institute of Subtropical Forestry, Chinese Academy of Forestry, Hangzhou, Zhejiang, People’s Republic of China

^2^Zhejiang Provincial Key Laboratory of Tree Breeding, Hangzhou, Zhejiang, People’s Republic of China

^3^Anhui Academy of Forestry, Hefei, Anhui, People’s Republic of China

*Corresponding author

E-mail: zczhou_risf@163.com (ZZ)

**Supplementary Information**

This file contains 3 supplementary figures，7 supplementary tables and captions of 2 supplementary files (Table S4 and Table S7).

**Supporting Figure Legends**

**Fig. S1. Functional annotation of assembled sequences based on gene ontology (GO) categorization.** The unigenes were annotated in three categories: cellular components, molecular functions and biological processes.

**Fig. S2. Clusters of orthologous group (COG) classification.**

**Fig. S3. Characterization of branches in the susceptible phenotype at 50 dpi; the needles turned yellow, and the tree died.**

**Fig. S1**


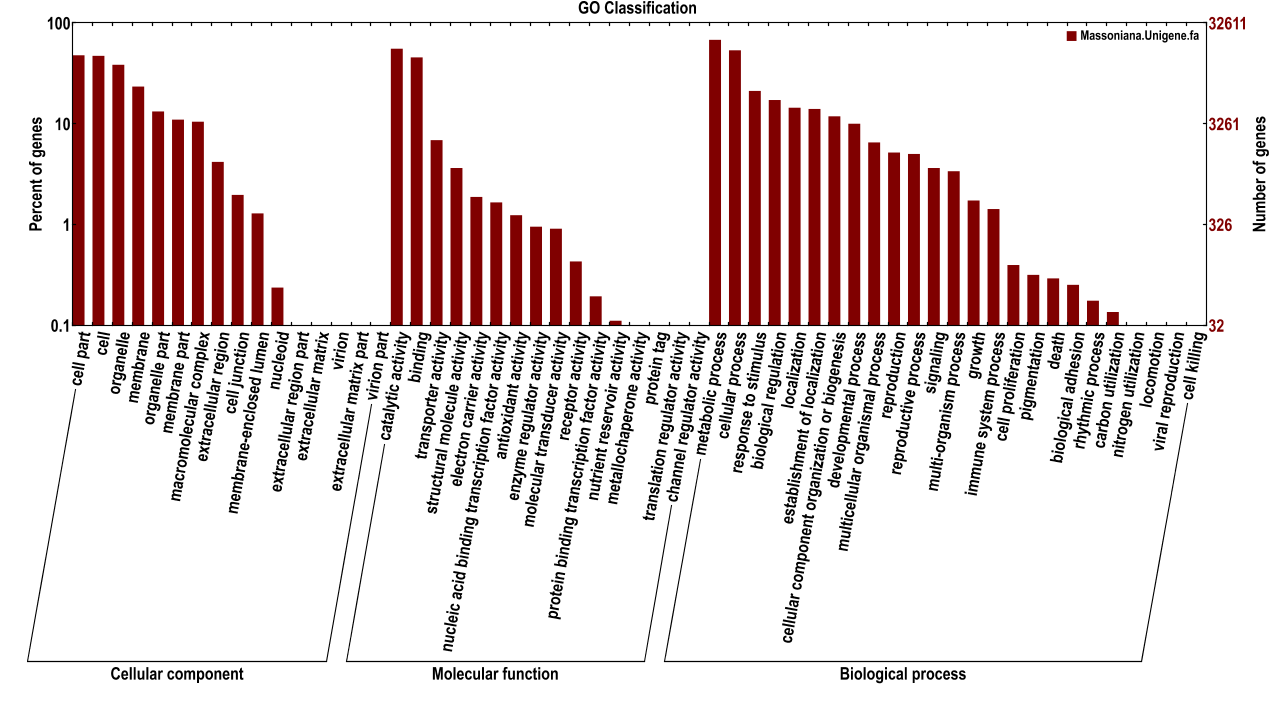


**Fig. S2**

**
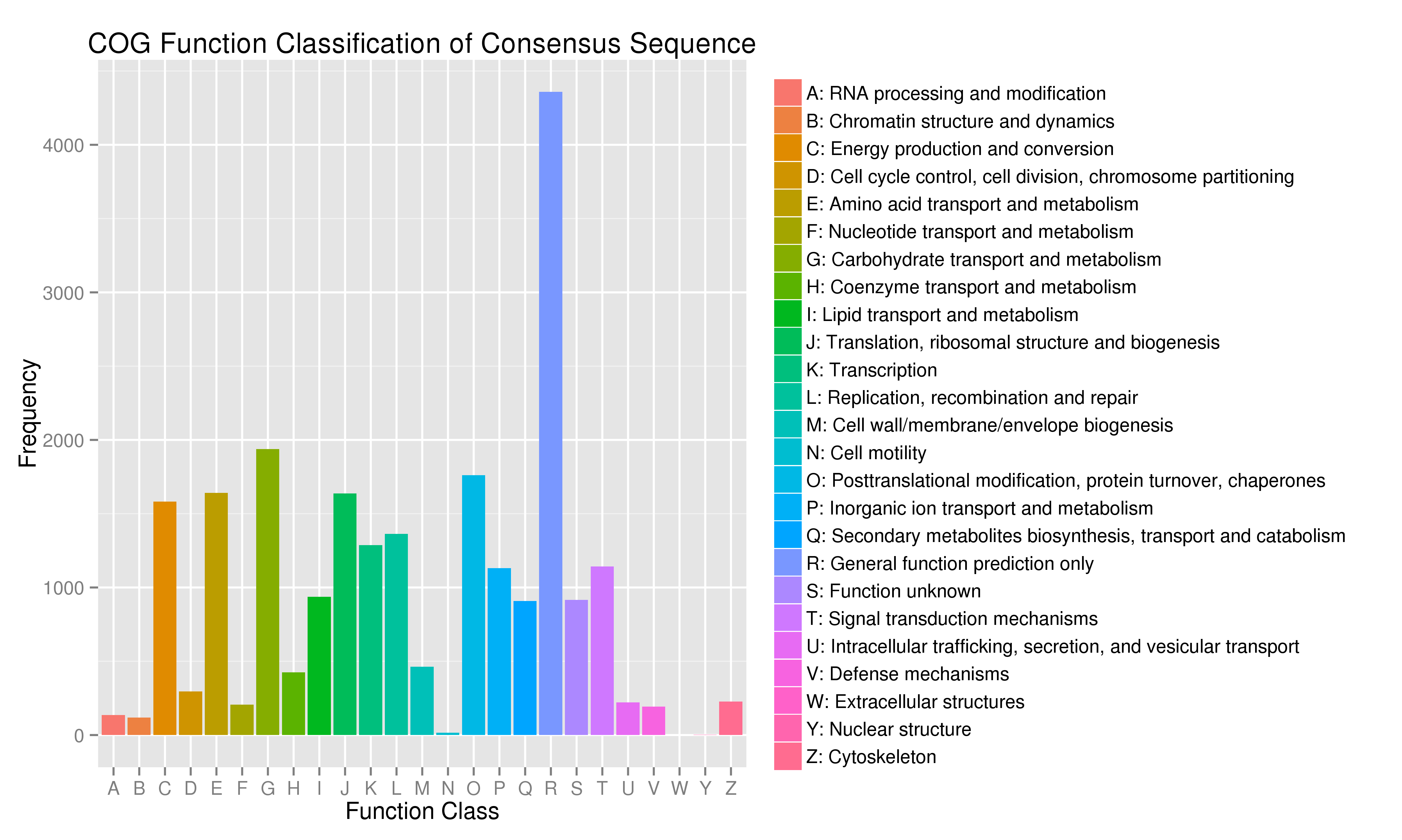
**

**Fig. S3**


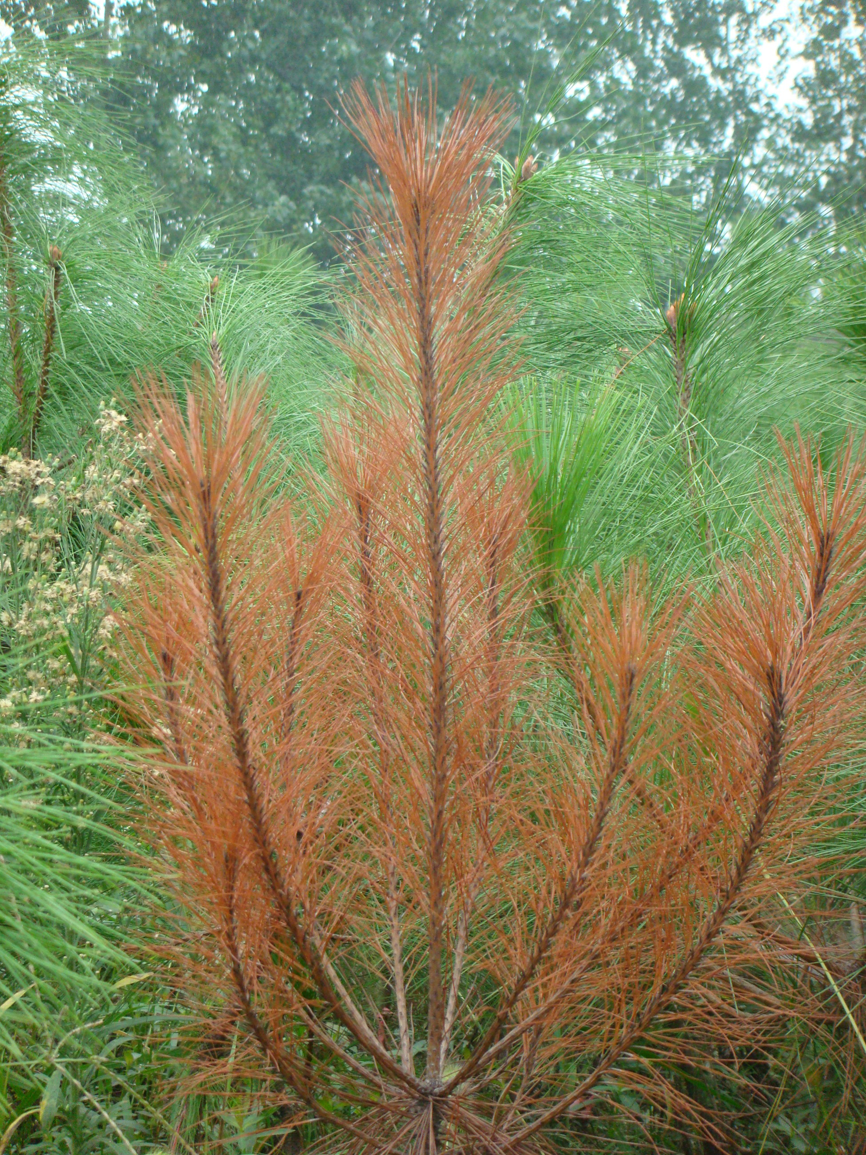


**Table S1. Summary of Illumina transcriptome assembly in masson pine.**

| **Nucleotide length (bp)** | **Contigs** | **Transcripts** | **Unigenes** |
| --- | --- | --- | --- |
| 0-300 | 7581034 | 41257 | 34372 |
| 300-500 | 22460 | 27067 | 18848 |
| 500-1000 | 14347 | 25183 | 12125 |
| 1000-2000 | 9723 | 23470 | 9216 |
| 2000+ | 5806 | 15669 | 5778 |
| Total number | 7633370 | 132648 | 80340 |
| Total length | 368145872 | 119978755 | 54267229 |
| N50 length | 48 | 1634 | 1245 |
| Mean length | 48.22849 | 904.49 | 675.4696 |

**Table S2. Correlations between 36 samples.**

|  | RI1-1 | RI1-2 | RI1-3 | RI15-1 | RI15-1 | RI15-3 | RI30-1 | RI30-2 | RI30-3 | RW1-1 | RW1-2 | RW1-3 | RW15-1 | RW15-2 | RW15-3 | RW30-1 | RW30-2 | RW30-3 | SI1-1 | SI1-2 | SI1-3 | SI15-1 | SI15-2 | SI15-3 | SI30-1 | SI30-2 | SI30-3 | SW1-1 | SW1-2 | SW1-3 | SW15-1 | SW15-2 | SW15-3 | SW30-1 | SW30-2 | SW30-3 |
| --- | --- | --- | --- | --- | --- | --- | --- | --- | --- | --- | --- | --- | --- | --- | --- | --- | --- | --- | --- | --- | --- | --- | --- | --- | --- | --- | --- | --- | --- | --- | --- | --- | --- | --- | --- | --- |
| RI1-1 | 1.00 | 1.00 | 0.99 | 0.93 | 0.92 | 0.89 | 0.90 | 0.90 | 0.86 | 0.99 | 0.99 | 0.98 | 0.88 | 0.92 | 0.91 | 0.87 | 0.76 | 0.87 | 0.97 | 0.97 | 0.98 | 0.83 | 0.84 | 0.88 | 0.86 | 0.87 | 0.87 | 0.95 | 0.97 | 0.97 | 0.89 | 0.89 | 0.89 | 0.85 | 0.83 | 0.85 |
| RI1-2 | 1.00 | 1.00 | 0.99 | 0.93 | 0.92 | 0.88 | 0.89 | 0.89 | 0.86 | 0.99 | 0.99 | 0.98 | 0.88 | 0.92 | 0.91 | 0.87 | 0.76 | 0.86 | 0.97 | 0.97 | 0.98 | 0.83 | 0.84 | 0.88 | 0.86 | 0.86 | 0.86 | 0.95 | 0.98 | 0.97 | 0.88 | 0.88 | 0.89 | 0.84 | 0.83 | 0.85 |
| RI1-3 | 0.99 | 0.99 | 1.00 | 0.92 | 0.90 | 0.86 | 0.86 | 0.87 | 0.84 | 0.98 | 0.99 | 0.98 | 0.87 | 0.91 | 0.90 | 0.84 | 0.74 | 0.83 | 0.97 | 0.96 | 0.98 | 0.82 | 0.83 | 0.85 | 0.81 | 0.82 | 0.83 | 0.95 | 0.97 | 0.96 | 0.86 | 0.86 | 0.86 | 0.82 | 0.80 | 0.82 |
| RI15-1 | 0.93 | 0.93 | 0.92 | 1.00 | 0.99 | 0.98 | 0.97 | 0.97 | 0.94 | 0.94 | 0.93 | 0.91 | 0.95 | 0.96 | 0.95 | 0.95 | 0.88 | 0.96 | 0.93 | 0.95 | 0.93 | 0.93 | 0.93 | 0.96 | 0.92 | 0.94 | 0.94 | 0.97 | 0.94 | 0.96 | 0.97 | 0.97 | 0.97 | 0.94 | 0.94 | 0.94 |
| RI15-1 | 0.92 | 0.92 | 0.90 | 0.99 | 1.00 | 0.98 | 0.98 | 0.98 | 0.96 | 0.93 | 0.91 | 0.89 | 0.95 | 0.96 | 0.96 | 0.96 | 0.89 | 0.96 | 0.92 | 0.94 | 0.91 | 0.94 | 0.94 | 0.97 | 0.92 | 0.94 | 0.94 | 0.97 | 0.93 | 0.95 | 0.97 | 0.97 | 0.97 | 0.95 | 0.95 | 0.95 |
| RI15-3 | 0.89 | 0.88 | 0.86 | 0.98 | 0.98 | 1.00 | 0.99 | 0.99 | 0.94 | 0.90 | 0.87 | 0.85 | 0.93 | 0.94 | 0.93 | 0.97 | 0.90 | 0.98 | 0.89 | 0.93 | 0.89 | 0.93 | 0.92 | 0.99 | 0.96 | 0.98 | 0.98 | 0.95 | 0.90 | 0.93 | 0.98 | 0.98 | 0.98 | 0.98 | 0.97 | 0.98 |
| RI30-1 | 0.90 | 0.89 | 0.86 | 0.97 | 0.98 | 0.99 | 1.00 | 1.00 | 0.97 | 0.91 | 0.88 | 0.85 | 0.92 | 0.94 | 0.92 | 0.99 | 0.91 | 0.99 | 0.90 | 0.93 | 0.89 | 0.92 | 0.91 | 0.98 | 0.96 | 0.98 | 0.98 | 0.95 | 0.91 | 0.94 | 0.98 | 0.98 | 0.98 | 0.97 | 0.96 | 0.97 |
| RI30-2 | 0.90 | 0.89 | 0.87 | 0.97 | 0.98 | 0.99 | 1.00 | 1.00 | 0.96 | 0.91 | 0.89 | 0.86 | 0.92 | 0.94 | 0.92 | 0.99 | 0.90 | 0.99 | 0.91 | 0.94 | 0.89 | 0.91 | 0.90 | 0.98 | 0.96 | 0.98 | 0.98 | 0.95 | 0.91 | 0.94 | 0.98 | 0.98 | 0.98 | 0.97 | 0.96 | 0.97 |
| RI30-3 | 0.86 | 0.86 | 0.84 | 0.94 | 0.96 | 0.94 | 0.97 | 0.96 | 1.00 | 0.87 | 0.86 | 0.82 | 0.91 | 0.90 | 0.90 | 0.96 | 0.92 | 0.95 | 0.87 | 0.89 | 0.86 | 0.94 | 0.93 | 0.95 | 0.91 | 0.93 | 0.93 | 0.93 | 0.88 | 0.91 | 0.95 | 0.95 | 0.95 | 0.92 | 0.94 | 0.92 |
| RW1-1 | 0.99 | 0.99 | 0.98 | 0.94 | 0.93 | 0.90 | 0.91 | 0.91 | 0.87 | 1.00 | 0.99 | 0.98 | 0.90 | 0.94 | 0.93 | 0.89 | 0.78 | 0.88 | 0.97 | 0.97 | 0.98 | 0.85 | 0.85 | 0.89 | 0.88 | 0.88 | 0.88 | 0.96 | 0.98 | 0.97 | 0.89 | 0.90 | 0.90 | 0.86 | 0.85 | 0.87 |
| RW1-2 | 0.99 | 0.99 | 0.99 | 0.93 | 0.91 | 0.87 | 0.88 | 0.89 | 0.86 | 0.99 | 1.00 | 0.99 | 0.89 | 0.93 | 0.92 | 0.87 | 0.77 | 0.86 | 0.98 | 0.97 | 0.99 | 0.84 | 0.85 | 0.86 | 0.83 | 0.84 | 0.85 | 0.95 | 0.98 | 0.97 | 0.87 | 0.88 | 0.87 | 0.84 | 0.83 | 0.84 |
| RW1-3 | 0.98 | 0.98 | 0.98 | 0.91 | 0.89 | 0.85 | 0.85 | 0.86 | 0.82 | 0.98 | 0.99 | 1.00 | 0.89 | 0.93 | 0.92 | 0.84 | 0.75 | 0.83 | 0.96 | 0.95 | 0.97 | 0.81 | 0.82 | 0.84 | 0.81 | 0.82 | 0.82 | 0.93 | 0.96 | 0.94 | 0.85 | 0.85 | 0.85 | 0.82 | 0.80 | 0.82 |
| RW15-1 | 0.88 | 0.88 | 0.87 | 0.95 | 0.95 | 0.93 | 0.92 | 0.92 | 0.91 | 0.90 | 0.89 | 0.89 | 1.00 | 0.97 | 0.96 | 0.91 | 0.93 | 0.92 | 0.88 | 0.89 | 0.89 | 0.93 | 0.93 | 0.90 | 0.86 | 0.89 | 0.88 | 0.91 | 0.88 | 0.89 | 0.89 | 0.90 | 0.90 | 0.91 | 0.92 | 0.91 |
| RW15-2 | 0.92 | 0.92 | 0.91 | 0.96 | 0.96 | 0.94 | 0.94 | 0.94 | 0.90 | 0.94 | 0.93 | 0.93 | 0.97 | 1.00 | 0.99 | 0.94 | 0.90 | 0.94 | 0.93 | 0.94 | 0.93 | 0.89 | 0.89 | 0.91 | 0.88 | 0.90 | 0.90 | 0.94 | 0.92 | 0.93 | 0.92 | 0.92 | 0.92 | 0.92 | 0.91 | 0.92 |
| RW15-3 | 0.91 | 0.91 | 0.90 | 0.95 | 0.96 | 0.93 | 0.92 | 0.92 | 0.90 | 0.93 | 0.92 | 0.92 | 0.96 | 0.99 | 1.00 | 0.92 | 0.88 | 0.92 | 0.91 | 0.92 | 0.91 | 0.89 | 0.89 | 0.90 | 0.87 | 0.89 | 0.89 | 0.93 | 0.91 | 0.92 | 0.91 | 0.91 | 0.91 | 0.90 | 0.90 | 0.90 |
| RW30-1 | 0.87 | 0.87 | 0.84 | 0.95 | 0.96 | 0.97 | 0.99 | 0.99 | 0.96 | 0.89 | 0.87 | 0.84 | 0.91 | 0.94 | 0.92 | 1.00 | 0.92 | 0.99 | 0.89 | 0.92 | 0.88 | 0.90 | 0.90 | 0.97 | 0.94 | 0.97 | 0.97 | 0.94 | 0.89 | 0.92 | 0.96 | 0.96 | 0.96 | 0.97 | 0.96 | 0.97 |
| RW30-2 | 0.76 | 0.76 | 0.74 | 0.88 | 0.89 | 0.90 | 0.91 | 0.90 | 0.92 | 0.78 | 0.77 | 0.75 | 0.93 | 0.90 | 0.88 | 0.92 | 1.00 | 0.93 | 0.78 | 0.81 | 0.77 | 0.89 | 0.87 | 0.87 | 0.83 | 0.87 | 0.85 | 0.83 | 0.78 | 0.80 | 0.85 | 0.86 | 0.86 | 0.90 | 0.92 | 0.89 |
| RW30-3 | 0.87 | 0.86 | 0.83 | 0.96 | 0.96 | 0.98 | 0.99 | 0.99 | 0.95 | 0.88 | 0.86 | 0.83 | 0.92 | 0.94 | 0.92 | 0.99 | 0.93 | 1.00 | 0.88 | 0.91 | 0.87 | 0.90 | 0.89 | 0.97 | 0.95 | 0.97 | 0.97 | 0.93 | 0.88 | 0.91 | 0.96 | 0.96 | 0.96 | 0.98 | 0.97 | 0.98 |
| SI1-1 | 0.97 | 0.97 | 0.97 | 0.93 | 0.92 | 0.89 | 0.90 | 0.91 | 0.87 | 0.97 | 0.98 | 0.96 | 0.88 | 0.93 | 0.91 | 0.89 | 0.78 | 0.88 | 1.00 | 0.99 | 1.00 | 0.85 | 0.86 | 0.90 | 0.85 | 0.87 | 0.88 | 0.97 | 0.99 | 0.98 | 0.90 | 0.90 | 0.90 | 0.87 | 0.85 | 0.87 |
| SI1-2 | 0.97 | 0.97 | 0.96 | 0.95 | 0.94 | 0.93 | 0.93 | 0.94 | 0.89 | 0.97 | 0.97 | 0.95 | 0.89 | 0.94 | 0.92 | 0.92 | 0.81 | 0.91 | 0.99 | 1.00 | 0.99 | 0.87 | 0.87 | 0.93 | 0.89 | 0.91 | 0.91 | 0.98 | 0.99 | 0.98 | 0.93 | 0.93 | 0.93 | 0.90 | 0.88 | 0.90 |
| SI1-3 | 0.98 | 0.98 | 0.98 | 0.93 | 0.91 | 0.89 | 0.89 | 0.89 | 0.86 | 0.98 | 0.99 | 0.97 | 0.89 | 0.93 | 0.91 | 0.88 | 0.77 | 0.87 | 1.00 | 0.99 | 1.00 | 0.85 | 0.85 | 0.88 | 0.84 | 0.86 | 0.86 | 0.97 | 0.99 | 0.98 | 0.89 | 0.89 | 0.88 | 0.86 | 0.84 | 0.86 |
| SI15-1 | 0.83 | 0.83 | 0.82 | 0.93 | 0.94 | 0.93 | 0.92 | 0.91 | 0.94 | 0.85 | 0.84 | 0.81 | 0.93 | 0.89 | 0.89 | 0.90 | 0.89 | 0.90 | 0.85 | 0.87 | 0.85 | 1.00 | 1.00 | 0.94 | 0.87 | 0.90 | 0.90 | 0.92 | 0.86 | 0.88 | 0.93 | 0.93 | 0.94 | 0.91 | 0.93 | 0.91 |
| SI15-2 | 0.84 | 0.84 | 0.83 | 0.93 | 0.94 | 0.92 | 0.91 | 0.90 | 0.93 | 0.85 | 0.85 | 0.82 | 0.93 | 0.89 | 0.89 | 0.90 | 0.87 | 0.89 | 0.86 | 0.87 | 0.85 | 1.00 | 1.00 | 0.94 | 0.85 | 0.89 | 0.89 | 0.93 | 0.87 | 0.89 | 0.92 | 0.92 | 0.93 | 0.89 | 0.92 | 0.89 |
| SI15-3 | 0.88 | 0.88 | 0.85 | 0.96 | 0.97 | 0.99 | 0.98 | 0.98 | 0.95 | 0.89 | 0.86 | 0.84 | 0.90 | 0.91 | 0.90 | 0.97 | 0.87 | 0.97 | 0.90 | 0.93 | 0.88 | 0.94 | 0.94 | 1.00 | 0.96 | 0.98 | 0.98 | 0.96 | 0.91 | 0.93 | 0.99 | 0.98 | 0.99 | 0.97 | 0.96 | 0.97 |
| SI30-1 | 0.86 | 0.86 | 0.81 | 0.92 | 0.92 | 0.96 | 0.96 | 0.96 | 0.91 | 0.88 | 0.83 | 0.81 | 0.86 | 0.88 | 0.87 | 0.94 | 0.83 | 0.95 | 0.85 | 0.89 | 0.84 | 0.87 | 0.85 | 0.96 | 1.00 | 0.99 | 0.99 | 0.91 | 0.86 | 0.90 | 0.94 | 0.94 | 0.95 | 0.95 | 0.93 | 0.96 |
| SI30-2 | 0.87 | 0.86 | 0.82 | 0.94 | 0.94 | 0.98 | 0.98 | 0.98 | 0.93 | 0.88 | 0.84 | 0.82 | 0.89 | 0.90 | 0.89 | 0.97 | 0.87 | 0.97 | 0.87 | 0.91 | 0.86 | 0.90 | 0.89 | 0.98 | 0.99 | 1.00 | 1.00 | 0.93 | 0.88 | 0.91 | 0.97 | 0.97 | 0.98 | 0.98 | 0.96 | 0.98 |
| SI30-3 | 0.87 | 0.86 | 0.83 | 0.94 | 0.94 | 0.98 | 0.98 | 0.98 | 0.93 | 0.88 | 0.85 | 0.82 | 0.88 | 0.90 | 0.89 | 0.97 | 0.85 | 0.97 | 0.88 | 0.91 | 0.86 | 0.90 | 0.89 | 0.98 | 0.99 | 1.00 | 1.00 | 0.93 | 0.88 | 0.92 | 0.97 | 0.97 | 0.97 | 0.98 | 0.96 | 0.98 |
| SW1-1 | 0.95 | 0.95 | 0.95 | 0.97 | 0.97 | 0.95 | 0.95 | 0.95 | 0.93 | 0.96 | 0.95 | 0.93 | 0.91 | 0.94 | 0.93 | 0.94 | 0.83 | 0.93 | 0.97 | 0.98 | 0.97 | 0.92 | 0.93 | 0.96 | 0.91 | 0.93 | 0.93 | 1.00 | 0.98 | 0.99 | 0.96 | 0.95 | 0.96 | 0.92 | 0.91 | 0.92 |
| SW1-2 | 0.97 | 0.98 | 0.97 | 0.94 | 0.93 | 0.90 | 0.91 | 0.91 | 0.88 | 0.98 | 0.98 | 0.96 | 0.88 | 0.92 | 0.91 | 0.89 | 0.78 | 0.88 | 0.99 | 0.99 | 0.99 | 0.86 | 0.87 | 0.91 | 0.86 | 0.88 | 0.88 | 0.98 | 1.00 | 0.99 | 0.91 | 0.91 | 0.91 | 0.88 | 0.86 | 0.88 |
| SW1-3 | 0.97 | 0.97 | 0.96 | 0.96 | 0.95 | 0.93 | 0.94 | 0.94 | 0.91 | 0.97 | 0.97 | 0.94 | 0.89 | 0.93 | 0.92 | 0.92 | 0.80 | 0.91 | 0.98 | 0.98 | 0.98 | 0.88 | 0.89 | 0.93 | 0.90 | 0.91 | 0.92 | 0.99 | 0.99 | 1.00 | 0.94 | 0.94 | 0.93 | 0.90 | 0.89 | 0.90 |
| SW15-1 | 0.89 | 0.88 | 0.86 | 0.97 | 0.97 | 0.98 | 0.98 | 0.98 | 0.95 | 0.89 | 0.87 | 0.85 | 0.89 | 0.92 | 0.91 | 0.96 | 0.85 | 0.96 | 0.90 | 0.93 | 0.89 | 0.93 | 0.92 | 0.99 | 0.94 | 0.97 | 0.97 | 0.96 | 0.91 | 0.94 | 1.00 | 1.00 | 0.99 | 0.96 | 0.94 | 0.95 |
| SW15-2 | 0.89 | 0.88 | 0.86 | 0.97 | 0.97 | 0.98 | 0.98 | 0.98 | 0.95 | 0.90 | 0.88 | 0.85 | 0.90 | 0.92 | 0.91 | 0.96 | 0.86 | 0.96 | 0.90 | 0.93 | 0.89 | 0.93 | 0.92 | 0.98 | 0.94 | 0.97 | 0.97 | 0.95 | 0.91 | 0.94 | 1.00 | 1.00 | 0.99 | 0.95 | 0.94 | 0.95 |
| SW15-3 | 0.89 | 0.89 | 0.86 | 0.97 | 0.97 | 0.98 | 0.98 | 0.98 | 0.95 | 0.90 | 0.87 | 0.85 | 0.90 | 0.92 | 0.91 | 0.96 | 0.86 | 0.96 | 0.90 | 0.93 | 0.88 | 0.94 | 0.93 | 0.99 | 0.95 | 0.98 | 0.97 | 0.96 | 0.91 | 0.93 | 0.99 | 0.99 | 1.00 | 0.95 | 0.94 | 0.95 |
| SW30-1 | 0.85 | 0.84 | 0.82 | 0.94 | 0.95 | 0.98 | 0.97 | 0.97 | 0.92 | 0.86 | 0.84 | 0.82 | 0.91 | 0.92 | 0.90 | 0.97 | 0.90 | 0.98 | 0.87 | 0.90 | 0.86 | 0.91 | 0.89 | 0.97 | 0.95 | 0.98 | 0.98 | 0.92 | 0.88 | 0.90 | 0.96 | 0.95 | 0.95 | 1.00 | 0.99 | 1.00 |
| SW30-2 | 0.83 | 0.83 | 0.80 | 0.94 | 0.95 | 0.97 | 0.96 | 0.96 | 0.94 | 0.85 | 0.83 | 0.80 | 0.92 | 0.91 | 0.90 | 0.96 | 0.92 | 0.97 | 0.85 | 0.88 | 0.84 | 0.93 | 0.92 | 0.96 | 0.93 | 0.96 | 0.96 | 0.91 | 0.86 | 0.89 | 0.94 | 0.94 | 0.94 | 0.99 | 1.00 | 0.99 |
| SW30-3 | 0.85 | 0.85 | 0.82 | 0.94 | 0.95 | 0.98 | 0.97 | 0.97 | 0.92 | 0.87 | 0.84 | 0.82 | 0.91 | 0.92 | 0.90 | 0.97 | 0.89 | 0.98 | 0.87 | 0.90 | 0.86 | 0.91 | 0.89 | 0.97 | 0.96 | 0.98 | 0.98 | 0.92 | 0.88 | 0.90 | 0.95 | 0.95 | 0.95 | 1.00 | 0.99 | 1.00 |

**Table S3. Functional annotation of the masson pine transcriptome.**

| **Annotated databases** | **All sequences** | **≥ 300 bp** | **≥ 1000 bp** |
| --- | --- | --- | --- |
| COG_Annotation | 16,489 | 5,592 | 6,032 |
| GO_Annotation | 32,611 | 11,608 | 10,461 |
| KEGG_Annotation | 10,259 | 3,634 | 3,549 |
| Swissprot_Annotation | 27,764 | 10,677 | 9,833 |
| nr_Annotation | 50,111 | 19,247 | 14,093 |
| Total | 50,227 | 19,293 | 14,102 |

**Table S5. Reads of 36 samples matched sequences in the transcriptome.**

| **Samples** | **Mapped Reads** | **Uniq mapped Reads** | **Multi mapped Reads** |
| --- | --- | --- | --- |
| RI-1-1 | 8,146,689 (82.85%) | 7,276,604 (89.32%) | 870,085 (10.68%) |
| RI-1-2 | 8,234,129 (82.11%) | 7,357,551(89.35%) | 876,578 (10.65%) |
| RI-1-3 | 9,015,088 (82.38%) | 8,056,067 (89.36%) | 959,021(10.64%) |
| RI-15-1 | 9,641,304 (83.62%) | 8,631,858 (89.53%) | 1,009,446 (10.47%) |
| RI-15-2 | 8,932,830 (83.93%) | 7,995,896 (89.51%) | 936,934 (10.49%) |
| RI-15-3 | 8,303,777 (82.02%) | 7,426,122 (89.43%) | 877,655 (10.57%) |
| RI-30-1 | 9,267,009 (84.28%) | 8,302,911 (89.60%) | 964,098 (10.40%) |
| RI-30-2 | 9,758,968 (83.81%) | 8,752,495 (89.69%) | 1,006,473 (10.31%) |
| RI-30-3 | 10,459,804 (85.87%) | 9,424,430 (90.10%) | 1,035,374 (9.90%) |
| RW-1-1 | 8,950,912 (81.54%) | 7,994,355 (89.31%) | 956,557 (10.69%) |
| RW-1-2 | 9,228,786 (82.29%) | 8,243,864 (89.33%) | 984,922 (10.67%) |
| RW-1-3 | 9,084,829 (80.99%) | 8,096,651 (89.12%) | 988,178 (10.88%) |
| RW-15-1 | 9,644,860 (83.00%) | 8,719,931 (90.41%) | 924,929 (9.59%) |
| RW-15-2 | 9,818,433 (82.35%) | 8,805,343 (89.68%) | 1,013,090 (10.32%) |
| RW-15-3 | 9,783,118 (82.53%) | 8,731,494 (89.25%) | 1,051,624 (10.75%) |
| RW-30-1 | 7,596,503 (83.71%) | 6,818,188 (89.75%) | 778,315 (10.25%) |
| RW-30-2 | 7,523,608 (83.60%) | 6,840,327 (90.92%) | 683,281 (9.08%) |
| RW-30-3 | 7,533,481 (83.41%) | 6,745,900 (89.55%) | 787,581 (10.45%) |
| SI-1-1 | 7,613,708 (81.31%) | 6,811,610 (89.47%) | 802,098 (10.53%) |
| SI-1-2 | 7,341,888 (81.73%) | 6,572,680 (89.52%) | 769,208 (10.48%) |
| SI-1-3 | 8,403,961 (81.98%) | 7,523,728 (89.53%) | 880,233 (10.47%) |
| SI-15-1 | 10,197,217 (84.02%) | 9,247,820 (90.69%) | 949,397 (9.31%) |
| SI-15-2 | 10,790,670 (84.18%) | 9,778,681 (90.62%) | 1,011,989 (9.38%) |
| SI-15-3 | 9,608,110 (83.64%) | 8,613,258 (89.65%) | 994,852 (10.35%) |
| SI-30-1 | 7,611,098 (82.55%) | 6,816,052 (89.55%) | 795,046 (10.45%) |
| SI-30-2 | 8,351,930 (83.23%) | 7,482,744 (89.59%) | 869,186 (10.41%) |
| SI-30-3 | 8,655,520 (83.71%) | 7,747,459 (89.51%) | 908,061 (10.49%) |
| SW-1-1 | 8,539,980 (81.86%) | 7,643,364 (89.50%) | 896,616 (10.50%) |
| SW-1-2 | 9,066,841 (80.47%) | 8,119,030 (89.55%) | 947,811 (10.45%) |
| SW-1-3 | 8,174,455 (80.70%) | 7,308,603 (89.41%) | 865,852 (10.59%) |
| SW-15-1 | 8,489,541 (81.84%) | 7,584,706 (89.34%) | 904,835 (10.66%) |
| SW-15-2 | 8,126,190 (83.10%) | 7,264,854 (89.40%) | 861,336 (10.60%) |
| SW-15-3 | 8,303,155 (81.96%) | 7,397,254 (89.09%) | 905,901 (10.91%) |
| SW-30-1 | 10,649,977 (80.76%) | 9,535,145 (89.53%) | 1,114,832 (10.47%) |
| SW-30-2 | 13,235,329 (84.35%) | 11,865,830 (89.65%) | 1,369,499 (10.35%) |
| SW-30-3 | 10,126,665 (80.70%) | 9,060,197 (89.47%) | 1,066,468 (10.53%) |

**Table S6. Number of DEGs and annotated DEGs.**

|  | **Num** | **Nr** | **SwissPort** | **GO** | **KEGG** | **COG** |
| --- | --- | --- | --- | --- | --- | --- |
| R1 | 260 | 217 | 150 | 126 | 40 | 58 |
| R15 | 371 | 314 | 217 | 195 | 60 | 88 |
| R30 | 152 | 112 | 73 | 63 | 16 | 31 |
| S1 | 756 | 642 | 368 | 333 | 126 | 270 |
| S15 | 2,179 | 1,636 | 1,038 | 946 | 320 | 613 |
| S30 | 398 | 338 | 199 | 189 | 70 | 108 |

**Table S8. The unigenes from DEGs were selected for qRT-PCR analysis.**

|  | ID | 2^-ΔΔCT | RNA |
| --- | --- | --- | --- |
| R_I1/W1_ | c50027.graph_c0 | 0.70 | 0.34 |
| R_I1/W1_ | c66357.graph_c0 | 0.21 | 0.38 |
| R_I1/W1_ | c66796.graph_c0 | 0.18 | 0.50 |
| R_I1/W1_ | c66887.graph_c0 | 0.49 | 0.14 |
| R_I1/W1_ | c68789.graph_c0 | 0.13 | 0.44 |
| R_I1/W1_ | c70340.graph_c0 | 2.55 | 2.51 |
| R_I1/W1_ | c75618.graph_c0 | 0.73 | 0.43 |
| R_I1/W1_ | c72778.graph_c0 | 1.96 | 2.64 |
| R_I1/W1_ | c77348.graph_c0 | 0.29 | 0.48 |
| R_I1/W1_ | c81022.graph_c0 | 31.43 | 44.60 |
| R_I15/W15_ | c62681.graph_c0 | 0.01 | 0.01 |
| R_I15/W15_ | c63172.graph_c0 | 12.67 | 14.80 |
| R_I15/W15_ | c75410.graph_c0 | 1.85 | 2.34 |
| R_I15/W15_ | c72881.graph_c0 | 4.16 | 3.61 |
| R_I30/W30_ | c34834.graph_c0 | 4.73 | 4.03 |
| R_I30/W30_ | c59150.graph_c0 | 1.83 | 2.01 |
| R_I30/W30_ | c64286.graph_c0 | 1.91 | 2.62 |
| R_I30/W30_ | c64286.graph_c1 | 4.74 | 2.56 |
| R_I30/W30_ | c75702.graph_c0 | 4.23 | 3.32 |
| S_I1/W1_ | c34454.graph_c0 | 3.99 | 4.20 |
| S_I1/W1_ | c44068.graph_c0 | 0.23 | 0.13 |
| S_I1/W1_ | c44874.graph_c0 | 0.28 | 0.14 |
| S_I1/W1_ | c47454.graph_c0 | 0.38 | 0.13 |
| S_I1/W1_ | c49924.graph_c0 | 0.36 | 0.17 |
| S_I1/W1_ | c75133.graph_c0 | 0.31 | 0.35 |
| S_I1/W1_ | c75410.graph_c0 | 0.46 | 0.47 |
| S_I1/W1_ | c78009.graph_c0 | 0.29 | 0.15 |
| S_I15/W15_ | c13604.graph_c0 | 0.10 | 0.07 |
| S_I15/W15_ | c31825.graph_c0 | 7.28 | 8.09 |
| S_I15/W15_ | c44068.graph_c0 | 0.31 | 0.27 |
| S_I15/W15_ | c44874.graph_c0 | 0.24 | 0.30 |
| S_I15/W15_ | c57095.graph_c0 | 0.21 | 0.20 |
| S_I15/W15_ | c60353.graph_c0 | 0.12 | 0.07 |
| S_I15/W15_ | c64867.graph_c0 | 0.36 | 0.26 |
| S_I15/W15_ | c65380.graph_c0 | 0.06 | 0.07 |
| S_I15/W15_ | c68079.graph_c0 | 0.21 | 0.13 |
| S_I15/W15_ | c69139.graph_c0 | 0.05 | 0.25 |
| S_I15/W15_ | c69878.graph_c1 | 0.22 | 0.25 |
| S_I15/W15_ | c74241.graph_c1 | 0.08 | 0.07 |
| S_I15/W15_ | c78009.graph_c0 | 0.27 | 0.29 |
| S_I15/W15_ | c82953.graph_c0 | 0.06 | 0.15 |
| S_I30/W30_ | c33611.graph_c0 | 0.26 | 0.40 |
| S_I30/W30_ | c55624.graph_c0 | 0.19 | 0.44 |
| S_I30/W30_ | c60547.graph_c0 | 0.44 | 0.48 |
| S_I30/W30_ | c64286.graph_c1 | 0.11 | 0.32 |
| S_I30/W30_ | c76015.graph_c0 | 0.23 | 0.45 |
| S_I30/W30_ | c77789.graph_c0 | 7.38 | 9.98 |
| S_I30/W30_ | c79812.graph_c0 | 0.01 | 0.01 |
| R_I1_/S_I1_ | c44068.graph_c0 | 6.04 | 4.40 |
| R_I1_/S_I1_ | c60353.graph_c0 | 1.83 | 1.21 |
| R_I1_/S_I1_ | c68079.graph_c0 | 5.37 | 4.95 |
| R_I1_/S_I1_ | c72881.graph_c0 | 3.98 | 4.66 |
| R_I1_/S_I1_ | c78009.graph_c0 | 7.03 | 3.18 |
| R_I15_/S_I15_ | c31825.graph_c0 | 0.28 | 0.18 |
| R_I15_/S_I15_ | c44068.graph_c0 | 3.58 | 2.57 |
| R_I15_/S_I15_ | c57095.graph_c0 | 12.72 | 7.78 |
| R_I15_/S_I15_ | c64867.graph_c0 | 7.54 | 7.10 |
| R_I15_/S_I15_ | c65380.graph_c0 | 3.89 | 3.19 |
| R_I15_/S_I15_ | c66357.graph_c0 | 3.01 | 2.43 |
| R_I15_/S_I15_ | c69878.graph_c0 | 14.23 | 6.99 |
| R_I15_/S_I15_ | c72778.graph_c0 | 4.73 | 3.65 |
| R_I15_/S_I15_ | c74241.graph_c1 | 5.50 | 3.41 |
| R_I15_/S_I15_ | c77348.graph_c0 | 2.75 | 3.00 |
| R_I15_/S_I15_ | c78009.graph_c0 | 3.61 | 2.36 |
| R_I15_/S_I15_ | c81022.graph_c0 | 6.36 | 5.21 |

**Table S9. Primers for quantitative real-time PCR.** Primers were designed from the sequences of the masson pine transcriptome library by using Primer Premier 3.0. Elongation factor 1-alpha (c60240.graph_c0) was used as the internal control.

| **Gene ID** | **Primers** | |
| --- | --- | --- |
| c113604.graph_c0 | F: CCTGAAATTCGACTGGCCCT | R: GCACCAGAGTCAGTGTTCGA |
| c31825.graph_c0 | F: TCACCCATCAAGAGAACCGA | R: GCAAGAAGCGGAATGGATAGC |
| c33611.graph_c0 | F: TTTTGTGCACTGACCGTTCG | R: CTGTTCCTTCGACGGCAGT |
| c34454.graph_c0 | F: AAGCCTTGAGTTGTGCAGCT | R: GGCCTTGAAAACATGGTTCCC |
| c34834.graph_c0 | F: AGTCCACCTGTCTTTTCGCA | R: ACAGAGGCGCTTACAGTGAC |
| c44068.graph_c0 | F: CCGGGATCAACAGAGGAAGA | R: GGTGGTGGAGAGCTCTTGTT |
| c44874.graph_c0 | F: TCCCTCATTGCTTTTCCCGG | R: TGCCAACCATATTCCAAAGCG |
| c47454.graph_c0 | F: TACACTGGACTCTACGGCGA | R: TAGCCAGAGCAGACCAGCTA |
| c49924.graph_c0 | F: GGCAACGTCAAGACTGATGG | R: ACGAATATCCACGTTGCGGT |
| c50027.graph_c0 | F: GGTCGATCGTCAATTGCTGC | R: CGTTTCGCAATGGATAGGGC |
| c55624.graph_c0 | F: GGCAACGTCAAGACTGATGG | R: ACGAATATCCACGTTGCGGT |
| c57095.graph_c0 | F: TCTGGGAGAAGGTTATGGG | R: CCTGGAGCAACAGAGGAAG |
| c59150.graph_c0 | F: CCTCCTTGAACCTGTGCCTT | R: CCTCCTTGAACCTGTGCCTT |
| c60353.graph_c0 | F: CTTCCGATCCTCCCATCGTG | R: TGAACGAGAAGGGCCACATC |
| c60547.graph_c0 | F: TAAATTCCAAGTGCCCCGCA | R: ACCGTGATACACATTTCAGA |
| c62681.graph_c0 | F: GAGTTAATCGGGCAGCGAGT | R: GCGGGTCGTAGTTGCAGATA |
| c63172.graph_c0 | F: TCACCACAACGATGCTGTCA | R: TATGGTGCCTGGATGTGCAG |
| c64286.graph_c0 | F: AACTTACCGCTCGGGGTTTT | R: CGATAAGCCTCGGCACTGAT |
| c64286.graph_c1 | F: ACGGATTTTCCTCTGACAGG | R: AAATGTTTGCCGCACTGAGG |
| c64867.graph_c0 | F: AAATCGTGTGTGTCCCTGCA | R: GGTTGCAATGATAACGGCCC |
| c65380.graph_c0 | F: AATGCATGCCCCAACGTTTC | R: GCTGGAATCGAGTGTCGGAT |
| c66357.graph_c0 | F: GTCGGTCGTCAGAATGGGTT | R: ATTGTCGACAGGCTTGGGAG |
| c66796.graph_c0 | F: GCATCCAAGTTGCCAAGCTC | R: TGACGCTCACTCTGATGGTG |
| c66887.graph_c0 | F: CTTTGCCTCGTCCATCACCT | R: GGTGAACGGATCCTGCATCA |
| c68789.graph_c0 | F: CGCCCGAATCTCTGCACTTA | R: TCGATGGTCTTGGTGATGGC |
| c69139.graph_c0 | F: CTGCCCAGCTGCTCCATAAT | R: ATCCGCAGACGTCACAAAGA |
| c69878.graph_c1 | F: CGCGATGACCAGAGCTAACT | R: ACTATGCTCGACAGGCTTGG |
| c70340.graph_c0 | F: GAGAGCACTCAGGACACCAG | R: GGAGGCTGGGTCGATAATGG |
| c72778.graph_c0 | F: CATGCGGATATGGCAGCCTA | R: GAGCTTGTAAAGGGCCGCTA |
| c72881.graph_c0 | F: GATCCGGGATGCTGTTGGAT | R: GGCGGGTGTCAATAGTTCCA |
| c74241.graph_c0 | F: CCAACGAACCTGACATCCGT | R: GAGATGGCTTCAGTGTTGCC |
| c74241.graph_c1 | F: TGGCTCTGGTTTCTGCTGTC | R: GAAACGGAGGTGGTCGAACT |
| c75133.graph_c0 | F: GCCCACATTTCAACCCACAC | R: GTGCCGGAGTGAACAACAAC |
| c75410.graph_c0 | F: ACGATATGGACGTCACGCTC | R: GGTCTGATGTCGCTGGTGAA |
| c75618.graph_c0 | F: ATGACCAGCAATCGTCCCAG | R: AAACCTCCCCTCGTTTTGCA |
| c75702.graph_c0 | F: TTGTTGTTGGCCGTTGCAAT | R: TAGGTAGGCATCTGGGCACT |
| c76015.graph_c0 | F: TACTACGGACGTGGACCCAT | R: GACACGGGGCGCTTATATGA |
| c77348.graph_c0 | F: CCGATGATGGAGACCTCAGC | R: CCTGTGAACGCACCCAAATG |
| c77789.graph_c0 | F: GCGAAGTGGGTGGAGTTACA | R: TCGGCAGCTTCCTAGACTCT |
| c78009.graph_c0 | F: TTGCGACTGACCCCAAACAT | R: TGGCGATCCACTTTGCTTCT |
| c79812.graph_c0 | F: TTTTGAGCCTCCTGCAGTCG | R: CCAGGCCTACGTGAATGGTT |
| c81022.graph_c0 | F: TTGGCTGTACAGATTCCCGT | R: ACCTATGGATGTCTGCTCCA |
| c82953.graph_c0 | F: ACTGTTAACCTGGCTCACGG | R: CTACGCAAATTCACCGCCAC |
| c60240.graph_c0 | F: AACGTCATTTCTAGCCGCCA | R: TCAGCCCTACAAACCCCTCT |

**Table S4. Details for 50,227 unigenes annotated in the xylem transcriptome in masson pine.**

**Table S7. DEGs were involved in the significantly enriched GO term “response to stress” in the susceptible phenotype at 1 dpi and 15 dpi.**
